# Overexpression of MIG-6 in the cartilage induces an osteoarthritis-like phenotype in mice

**DOI:** 10.1186/s13075-020-02213-z

**Published:** 2020-05-19

**Authors:** Melina Bellini, Michael A. Pest, Manuela Miranda-Rodrigues, Ling Qin, Jae-Wook Jeong, Frank Beier

**Affiliations:** 1 Department of Physiology and Pharmacology, Western University, London, ON Canada; 2 Western University Bone and Joint Institute, London, ON Canada; 3 Children’s Health Research Institute, London, ON Canada; 4 Department of Orthopaedic Surgery, Perelman School of Medicine, University of Pennsylvania, Philadelphia, PA USA; 5 Department of Obstetrics, Gynecology and Reproductive Biology, Michigan State University College of Human Medicine, Grand Rapids, MI USA

**Keywords:** Mitogen inducible gene-6, Epidermal growth factor receptor, Osteoarthritis, Articular cartilage

## Abstract

**Background:**

Osteoarthritis (OA) is the most common form of arthritis and characterized by degeneration of the articular cartilage. Mitogen-inducible gene 6 (Mig-6) has been identified as a negative regulator of the epidermal growth factor receptor (EGFR). Cartilage-specific *Mig-6* knockout (KO) mice display increased EGFR signaling, an anabolic buildup of the articular cartilage, and formation of chondro-osseous nodules. Since our understanding of the EGFR/Mig-6 network in the cartilage remains incomplete, we characterized mice with cartilage-specific overexpression of Mig-6 in this study.

**Methods:**

Utilizing knee joints from cartilage-specific *Mig-6-*overexpressing (*Mig-6*^*over/over*^) mice (at multiple time points), we evaluated the articular cartilage using histology, immunohistochemical staining, and semi-quantitative histopathological scoring (OARSI) at multiple ages. MicroCT analysis was employed to examine skeletal morphometry, body composition, and bone mineral density.

**Results:**

Our data show that cartilage-specific *Mig-6* overexpression did not cause any major developmental abnormalities in the articular cartilage, although *Mig-6*^*over/over*^ mice have slightly shorter long bones compared to the control group. Moreover, there was no significant difference in bone mineral density and body composition in any of the groups. However, our results indicate that *Mig-6*^*over/over*^ male mice show accelerated cartilage degeneration at 12 and 18 months of age. Immunohistochemistry for SOX9 demonstrated that the number of positively stained cells in *Mig-6*^*over/over*^ mice was decreased relative to controls. Immunostaining for MMP13 appeared increased in areas of cartilage degeneration in *Mig-6*^*over/over*^ mice. Moreover, staining for phospho-EGFR (Tyr-1173) and lubricin (PRG4) was decreased in the articular cartilage of *Mig-6*^*over/over*^ mice.

**Conclusion:**

Overexpression of *Mig-6* in the articular cartilage causes no major developmental phenotype; however, these mice develop earlier OA during aging. These data demonstrate that Mig-6/EGFR pathways are critical for joint homeostasis and might present a promising therapeutic target for OA.

## Introduction

Osteoarthritis (OA), a chronic degenerative joint disease, is the most common form of arthritis. OA affects 242 million individuals worldwide, but that number will grow due to increasing life expectancies [1]. This statistic is alarming, considering the disability, the loss of quality of life, and the costs to the health system generated by OA. Currently, there are pharmacological treatments available to manage OA symptoms such as pain [2–4] as well as surgical joint replacement at the end stage of disease [5, 6]. Unfortunately, however, there is no cure for OA. Progressive understanding of the pathophysiology of OA suggests that the disease is a heterogeneous condition, so further research is needed to direct the clinical approaches to disease management [7].

Recent studies have shown that OA is a multifactorial disease of the whole joint; however, its pathogenesis remains still poorly understood [8]. Genetic, environmental, and biomechanical factors can accelerate the onset of OA [9]. The articular cartilage is a highly specialized tissue that forms the smooth gliding surface of synovial joints, with chondrocytes as the only cellular component of the cartilage [10]. The homeostasis of the cartilage extracellular matrix (ECM) involves a dynamic equilibrium between anabolic and catabolic pathways controlled by chondrocytes [11]. The progression of OA is associated with dramatic alteration in the integrity of the cartilage ECM network formed by a large number of proteoglycans (mostly aggrecan), collagen II, and other non-collagenous matrix proteins [12]. In addition, ECM synthesis is regulated by a number of transcriptional regulators involved in chondrogenesis, specifically sex-determining- region-Y box 9 (SOX9), L-SOX 5, and SOX6 that regulate type II collagen (*Col2a1*) and aggrecan (*Acan*) gene expression [13]. On the other hand, catabolic events are dominant in OA, and cells are exposed to degenerative enzymes such as aggrecanases (e.g., ADAMTS-4, ADAMTS-5) [12, 14], collagenases (e.g., MMP-1, MMP-3, MMP-8, MMP-13) [15], and gelatinases (e.g.,MMP-2 and MMP-9), all of which have implications in articular cartilage degeneration [16]. A number of growth factors [17] play a role in OA pathology, such as transforming growth factor-β [18], BMP-2 [19], insulin growth factor 1 (IGF-1) [20], and fibroblast growth factor (FGF), but the exact regulation of chondrocyte physiology is still not completely understood.

Recent studies in our laboratory [21, 22] have identified the epidermal growth factor receptor (EGFR) and its ligand transforming growth factor alpha (TGFα) as possible mediators of cartilage degeneration [23–25]. The human *TGFA* gene locus was also strongly linked to hip OA and cartilage thickness in genome-wide association studies [26, 27]. TGFα stimulates EGFR signaling and activates various cell-signaling pathways in chondrocytes, including extracellular signal-regulated kinase 1 and 2 (ERK1/2) and phosphoinositide 3-kinase (P13K) [28]. EGFR signaling plays important roles in endochondral ossification [29, 30], growth plate development [29], and cartilage maintenance and homeostasis [31–33], but many aspects of its action in the cartilage are still not well understood. However, both protective and catabolic effects of EGFR signaling in OA have been reported, suggesting context-specific roles of this pathway [34].

Mitogen-inducible gene 6 (Mig-6) is also known as Gene 33, ErbB receptor feedback inhibitor 1 (ERRFI1), or RALT and is found in the cytosol [35]. *Mig-6* protein binds to and inhibits EGFR signaling through a two-tiered mechanism: suppression of EGFR catalytic activity and receptor downregulation [36]. Interestingly, various studies have reported that loss of Mig-6 induces the onset of OA-like symptoms in mice [35, 37–39]. Cartilage-specific (Col2-Cre) knockout of *Mig-6* mice results in the formation of chondro-osseous nodules in the knee, but also increased thickness of the articular cartilage in the knee, ankle, and elbow [40]. *Prx1*-Cre-mediated knockout of *Mig-6* in the limb mesenchyme results in a similar phenotype as that observed in cartilage-specific knockout mice [32]. These phenotypes appeared to be caused by an increase in chondrocyte proliferation in articular cartilage, supported by the increased expression of Sox9 and EGFR activation in the cartilage [32]. Since our studies suggest dosage- and/or context-specific roles of EGFR signaling in the process of cartilage degeneration in OA, in this study, we used a cartilage-specific (Col2-Cre) to examine effects of Mig-6 overexpression specifically in articular cartilage. We hypothesized that overexpression of Mig-6/EGFR accelerates cartilage degeneration during aging.

## Materials and methods

### Generation of Mig-6 overexpression mice

*Mig-6* overexpression animals on a mixed C57Bl/6 and agouti mouse background, with the overexpression cassette in the Rosa26 locus [41], and bred for 10 generations into a C57Bl/6 background were used. Transcription of *Mig-6* is under the control of a ubiquitously expressed chicken beta actin-cytomegalovirus hybrid (CAGGS) promoter but blocked by a “Stop Cassette” flanked by LoxP sites (LSL) [41]. *Mig-6* overexpression mice were bred to mice carrying the Cre recombinase gene under the control of the Collagen 2 promoter [42], to induce recombination and removal of the Stop Cassette specifically in the cartilage. Throughout the manuscript, animals for homozygote overexpression of Mig-6 from both alleles are termed *Mig-6*^*over/over*^ (*Mig-6*^*over/over*^*Col2a1-Cre*^*+/−*^), while control mice are identical but without the Cre gene (noted as “control” in this manuscript for simplicity). Mice were group-housed (at least one pair of littermate-matched control and overexpression animals), on a standard 12-h light/dark cycle, without access to running wheels and with free access to mouse chow and water. Animals were weighed prior to euthanasia by asphyxiation with CO_2_. All animal experiments were done in accordance with the Animal Use Subcommittee at the University of Western Ontario and conducted in accordance with the guidelines from the Canadian Council on Animal Care.

### Genotyping

Genotype was determined by polymerase chain reaction (PCR) analysis using DNA processed from biopsy samples of ear tissue from mice surviving to at least 21 days of age. PCR strategy: primer set P1 and P2 can amplify a 300-bp fragment from the wild-type allele, whereas P1 and P3 can amplify a 450-bp fragment from the targeted ROSA26 locus allele [41] (Supplementary Figure/Table 1).

### RNA isolation and quantitative real-time PCR

Total RNA was isolated from postnatal day 0 (P0) mouse cartilage of *Mig-6*^*over/over*^ and control littermates using TRIzol® (Invitrogen) as per the manufacturer’s instructions and as previously described [43]. Complementary DNA (cDNA) was synthesized using the iScript cDNA Synthesis kit (Bio-Rad) with 1 μg of RNA (Bio-Rad Laboratories) and combined with 300 nM of forward and reverse primers (for primer sequences, please see Supplementary Figure 1E) as well as iQ™ SYBR® Green Supermix (Bio-Rad Laboratories) for PCR on a Bio-Rad CFX384 RT-PCR system. Relative gene expression was normalized to the internal control glyceraldehyde 3-phosphate dehydrogenase (*Gapdh*), calculated using the ΔΔCT method.

### Histopathology of the knee

The limbs from *Mig-6*^*over/over*^ and control mice were harvested and fixed in 4% paraformaldehyde (Sigma) for 24 h and decalcified in ethylenediaminetetraacetic acid (5% EDTA in phosphate-buffered saline (PBS), pH 7.0. The joints were processed and embedded in paraffin in sagittal or frontal orientation, with the serial sections taken at a thickness of 5 μm. Sections were stained with toluidine blue (0.04% toluidine blue in 0.2 M acetate buffer, pH 4.0, for 10 min) for glycosaminoglycan content and general evaluation of the articular cartilage. All images were taken with a Leica DFC295 digital camera and a Leica DM1000 microscope.

### Thickness of proximal tibia growth plate

For early developmental time points such as newborn (P0), the sagittal knee sections stained with toluidine blue were used to measure the width of the zones of the epiphyseal growth plate in the proximal tibia. The average thickness of the resting and proliferative zones combined was evaluated by taking three separate measurements at approximately equal intervals across the width of the growth plate. The average hypertrophic zone thickness was also measured using 3 different measurements across the width of the growth plate, starting each measurement at the border of the proliferative and hypertrophic zones and ending at the subchondral bone interface. The third average measurement was then taken for the thickness of the entire growth plate. The ImageJ software (v.1.51) [44] was used for all measures, with the observer blinded to the genotype.

### Articular cartilage evaluation

Articular cartilage thickness was measured from toluidine blue-stained frontal sections by a blinded observer. Articular cartilage thickness was measured separately for the non-calcified articular cartilage (measured from the superficial tangential zone to the tidemark), and the calcified articular cartilage (measured from the subchondral bone to the tidemark) across three evenly spaced points from all four quadrants of the joint (medial/lateral tibia and femur) in 4 sections spanning at least 500 μm. The ImageJ software (v.1.51) [44] was used to measure the thickness of the articular cartilage.

### Micro-computerized tomography (μCT)

Whole-body scans were collected in 6-week-, 11-week-, 12-month-, and 18-month-old control and *Mig-6*^*over/over*^ male and female mice. Mice were euthanized and imaged using the General Electric (GE) SpeCZT microCT machine [45] at a resolution of 50 μm/voxel or 100 μm/voxel. GE Healthcare MicroView software (v2.2) was used to generate 2D maximum intensity projection and 3D isosurface images to evaluate skeletal morphology. MicroView was used to create a line measurement tool in order to calculate the bone lengths; femur lengths were calculated from the proximal point of the greater trochanter to the base of the lateral femoral condyle. Tibia lengths were measured from the midpoint medial plateau to the medial malleolus. Humerus lengths were measured from the midpoint of the greater tubercle to the center of the olecranon fossa.

### Body composition analysis

MicroView software (GE Healthcare Biosciences) was used to analyze the microCT scans at the resolution of 100um/voxel. Briefly, the region of interest (ROI) was used to calculate the mean of air, water, and an epoxy-based, cortical bone-mimicking calibrator (SB3; Gammex, Middleton, WI, USA) (1100 mg/cm^3^) [46]. A different set of global thresholds was applied to measure adipose, lean, and skeletal mass (− 275, − 40, and 280 Hounsfield units (HU), respectively). Moreover, bone mineral density (BMD) was acquired as the ratio of the average HU (from the value of the skeletal region of interest) in order to calculate the HU value of the SB3 calibrator, multiplied by the known density of the SB3 as described [45].

### OARSI histopathology scoring

Serial sections through the entire knee joint were scored according to the OARSI histopathology scoring system [47] by two blinded observers on the four quadrants of the knee: lateral femoral condyle (LFC), lateral tibial plateau (LTP), medial femoral condyle (MFC), and medial tibial plateau (MTP). Histologic scoring from 0 to 6 represent the OA severity, from 0 (healthy cartilage) to 6 (erosion of more than 75% of the articular cartilage). Individual scores are averaged first for each observer, then across observers, and OA severity is shown as described for each graph. Scores were compared between male and female *Mig-6*^*over/over*^ and control mice at both 12 and 18 months of age. All images were taken with a Leica DFC295 digital camera and a Leica DM1000 microscope.

### Immunohistochemistry

Frontal paraffin sections of knees were used for immunohistochemical analysis, with slides with “no primary antibody” as a control. All sections were deparaffinized and rehydrated as previously described [40, 48]. Subsequently, the sections were incubated in 3% H_2_O_2_ in methanol for 15 min to inhibit endogenous peroxidase activity. After rinsing with water, 5% goat or donkey serum in PBS was applied to reduce non-specific background staining. The sections were incubated overnight at 4 °C with primary antibodies against SOX9 (R&D Systems, AF3075), MMP13 (Protein Tech, Chicago, IL, USA, 18165-1-AP), lubricin (Abcam, ab28484) (primary concentration antibody for all three 1:100), and phospho-EGFR (phosphoTyr-1173; Cell Signaling Technology) (primary concentration antibody 1:50). After washing, the sections were incubated with horseradish peroxidase (HRP)-conjugated donkey anti-goat or goat anti-rabbit secondary antibody (R&D System and Santa Cruz, secondary concentration antibody 1:200), before incubation with diaminobenzidine substrate as a chromogen (Dako, Canada). Finally, the sections were counterstained with 0.5% methyl green (Sigma) and mounted. Cell density of articular cartilage chondrocytes from 6- and 11-week-old male mice was determined by counting all lacunae with evidence of nuclear staining in the lateral and medial femur/tibia using a centered region of interest measuring 200 μm wide and 70 μm deep from the articular surface by a blinded observer. For newborn (P0) animals, the region of interest was measured 200 μm wide and 100 μm deep from the proliferative zone.

### Statistical analysis

All statistical analyses were performed using GraphPad Prism (v6.0). Differences between the two groups were evaluated using Student’s *t* test, and two-way ANOVA was used to compare the 4 groups followed by a Bonferroni multiple comparisons test. All *n* values represent the number of cartilage-specific *Mig-6-*overexpressing mice and control littermates used in each group.

## Results

### Increased MIG-6 mRNA from overexpression of *Mig-6* mice and decreased EGFR staining

**Fig. 1:**
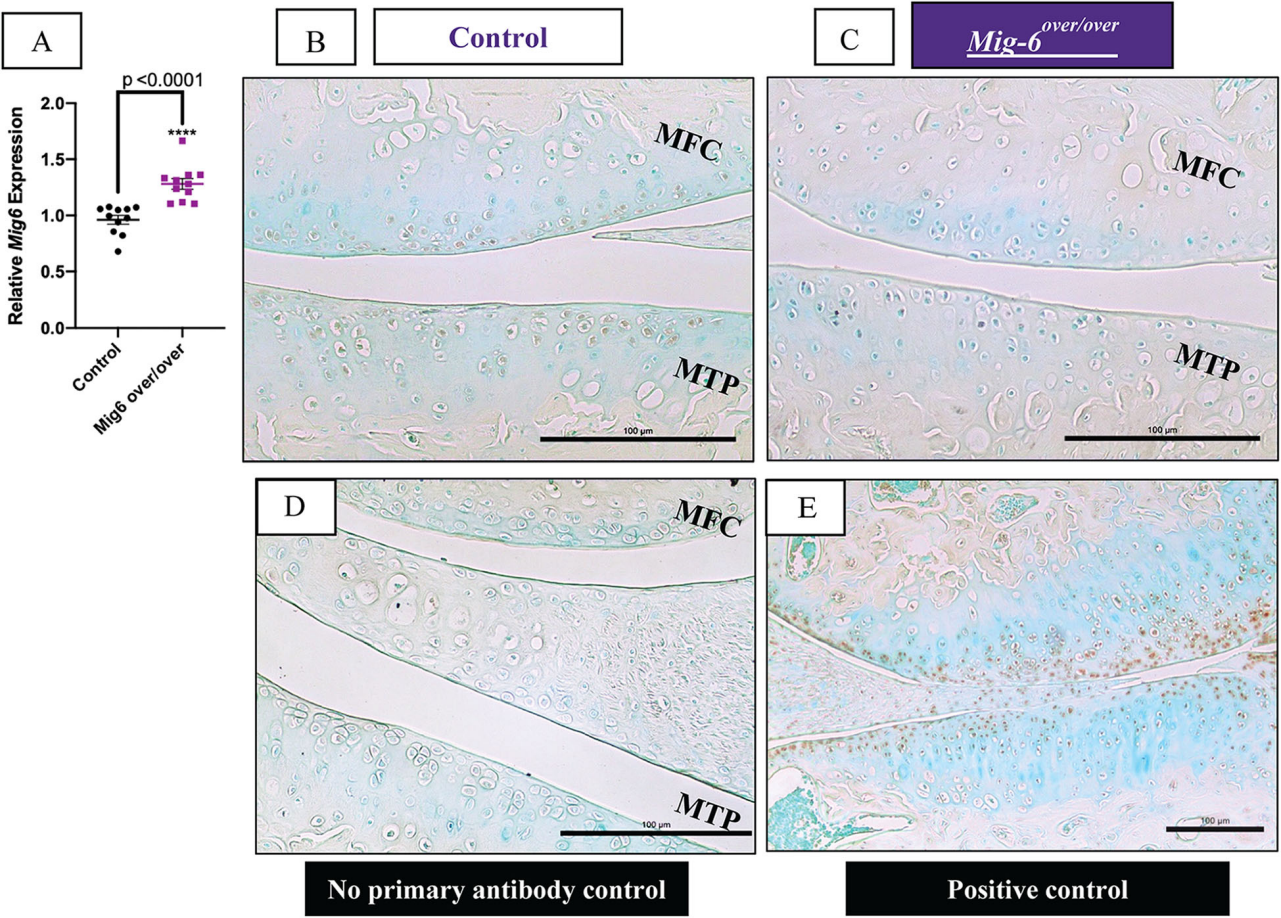
Mig-6 mRNA and EGFR protein expression in the articular cartilage of cartilage-specific Mig-6-overexpressing mice. **a** Levels of Mig-6 mRNA determined by qRT-PCR analysis on P0 epiphysis. Individual data points presented with mean ± SEM (*P* < 0.05). Data analyzed by two-tailed Student *t* tests from 11 mice per group. **b** Immunostaining of phosphorylated epidermal growth factor receptor (pEGFR; Tyr-1173) in the knee joints of 11-week-old control and **c***Mig-6*^*over/over*^ (*Mig-6*^*over/over*^*Col2a1-Cre*^*+/−*^) is decreased in response to increased Mig-6 levels. Frontal sections of mouse articular cartilage incubated without primary antibody, as a negative control, exhibited no staining (**d**). Cartilage-specific Mig-6 KO mice served as a positive control (**e**). *N* = 5 mice/genotyping. MFC, medial femoral condyle; MTP, medial tibial plateau. Scale bar = 100 μm

We bred mice for conditional overexpression of Mig-6 [41] to mice expressing Cre recombinase under the control of the collagen II promoter. Homozygote mice overexpressed Mig-6 in all collagen II-producing cells (and their progeny) from both Rosa26 alleles and are referred to as *Mig-6*^*over/over*^ from here on. Control mice do not express Cre. Genomic DNA was extracted from ear notches to identify homozygous mice *Mig-6*^*over/over*^using standard PCR analysis. Overexpressing mice were obtained at the expected Mendelian ratios (data not shown). Real-time RT-PCR revealed increased levels of *Mig-6* expression in the cartilage of *Mig-6*^*over/over*^ mice from postnatal day 0 (PO) (Fig. 1a). Since Mig-6 negatively regulates EGFR signaling [31, 32, 40], immunohistochemistry was performed for phospho-EGFR (Tyr-1173) (pEGFR), with no primary antibody controls. Frontal knee sections from 11-week-old male *Mig-6*^*over/over*^ mice showed decreased pEGFR staining in the medial compartment in the knee joint (Fig. 1b–e), as expected upon Mig-6 overexpression.

### Overexpression of Mig-6 has minor effects on skeletal phenotypes during development

Male mutant gained weight at the same rate as controls over the examined 10-week period, while female *Mig-6*^*over/over*^ mice were slightly lighter than controls starting at 8 weeks of age (Suppl. Fig. 2A-B). These differences persisted at 12 months of age for female mice, while at 18 months, both male and female mutant mice were lighter than their controls (Suppl. Fig. 2C-D). Growth plates of postnatal day 0 (P0) *Mig-6*^*over/over*^ and control mice were analyzed by histology. No major differences in the tibia growth plate architecture were seen between genotypes (Suppl. Fig. 3A). While the length of the total growth plate was slightly reduced in *Mig-6*^*over/over*^ mice, differences in lengths of either the combined resting/proliferative or hypertrophic zones were not statistically significant (Suppl. Fig. 3B-D).

### Mice overexpressing Mig-6 have shorter long bones than control mice

**Fig. 2:**
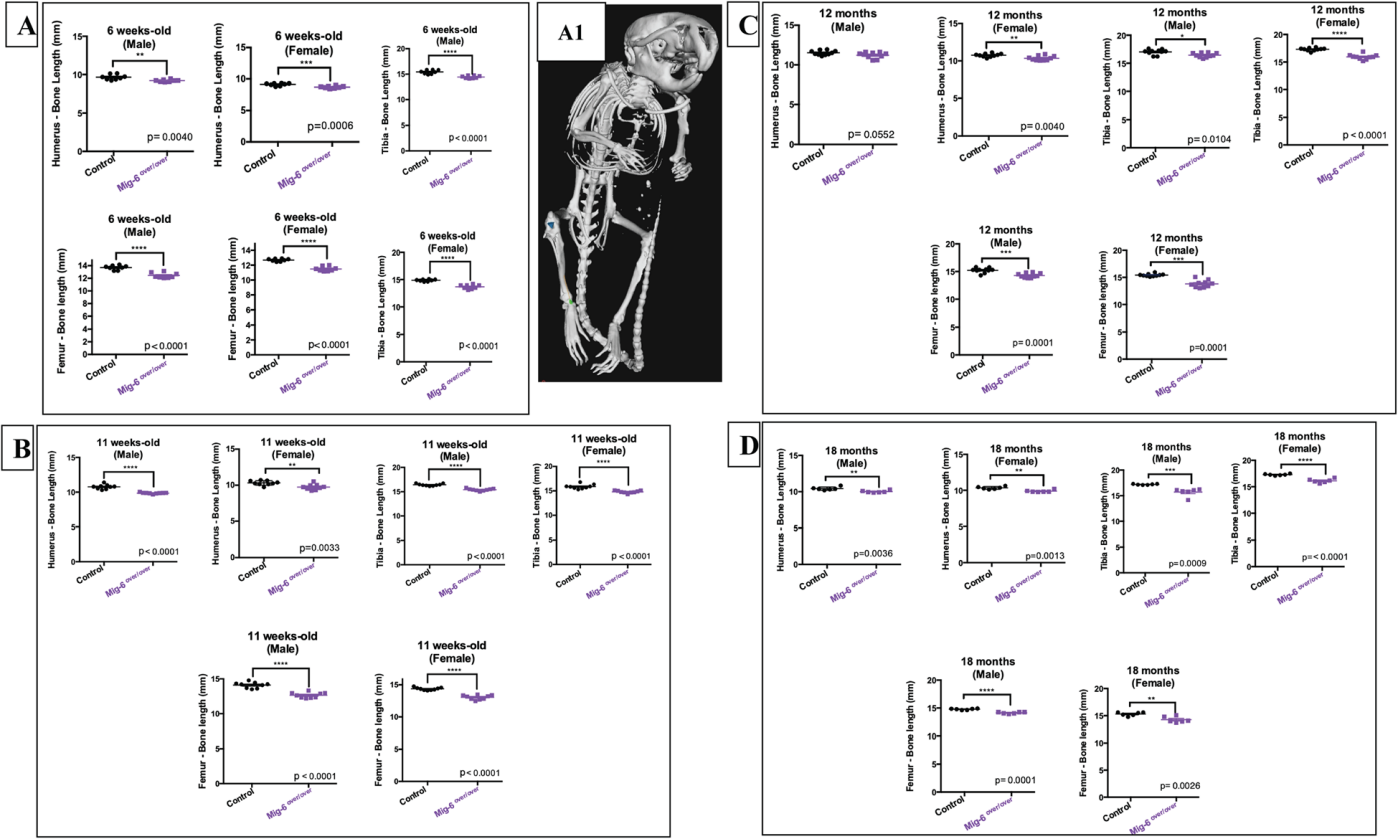
Long bones of Mig-6-overexpressing mice are significantly shorter than those of control long bone during growth and aging. The lengths of the right humeri, tibiae, and femora were measured on microCT scans of mice at different ages using GE MicroView software. **a** 6-week-old male and female control and Mig-6-overexpressing mice. **b** Eleven-week-old male and female control and Mig-6-overexpressing mice. **c** Twelve-month-old male and female control and Mig-6-overexpressing mice. **d** Eighteen-month-old male and female control and Mig-6-overexpressing mice. (A1) Representative 3D isosurface reconstructions of 100 μm/voxel μCT scans. There were statistically significant differences between control and Mig-6^over/over^ male and female groups. Individual data points presented with mean ± SEM (*P* < 0.05). Data analyzed by two-tailed Student *t* tests from 6 to 12 mice per group (age/gender)

Skeletal morphology and bone length were examined by microCT mice at the ages of 6 weeks and 12 and 18 months. Scans of *Mig-6*^*over/over*^ male and female mice and their controls were used to generate 3D isosurface reconstructions of 100 μm/voxel μCT scans, in order to measure long bone lengths (femurs, humeri, and tibiae) in GE MicroView v2.2 software. Mutant bones were slightly shorter throughout life, with the exception of the male humeri at 12 months that did not show any statistically significant difference (Fig. 2a–d). In contrast, male mice did not show any differences in bone mineral density at 11 weeks, 12 months, or 18 months, compared to controls (Suppl. Fig. 4A-C). In addition, no differences in body mass composition were seen in male mutant and control mice at 11 weeks, 12 months, and 18 months of age (Suppl. Fig. 5A1-C2).

### Mig-6-overexpressing mice have healthy articular cartilage during skeletal maturity

**Fig. 3:**
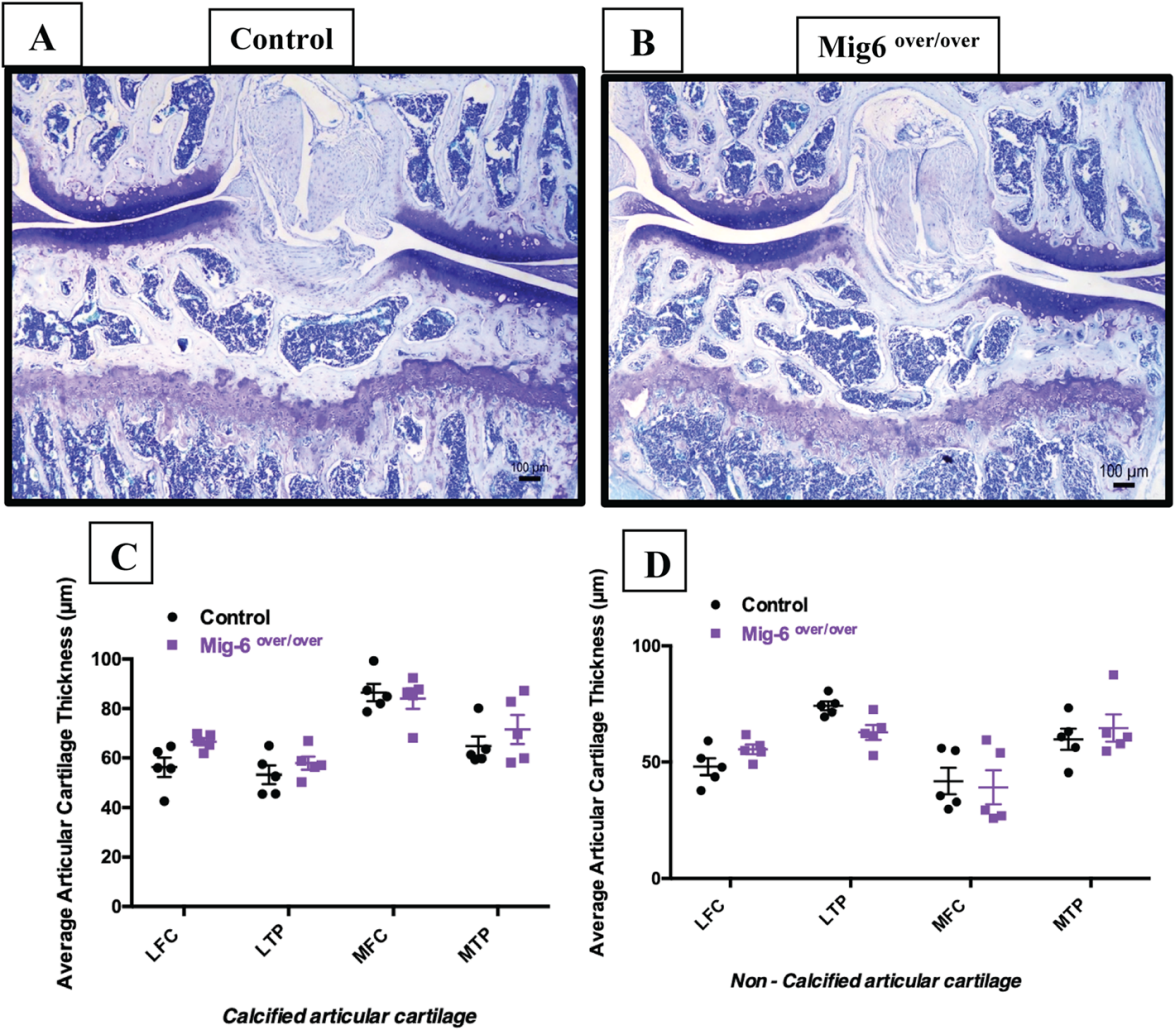
Articular cartilage from 11-week-old *Mig-6*^*over/over*^ male mice appeared healthy at skeletal maturity. Representative (*n* = 5/group, toluidine blue) stained frontal sections of the knee joints from 11-week-old control (**a**) and *Mig-6*^*over/over*^ (**b**) mice. Mig-6-overexpressing mice show similar articular cartilage thickness when compared to controls at 11 weeks of age. The average thickness of the calcified articular cartilage (**c**) and non-calcified articular cartilage (**d**) in the lateral femoral condyle (LFC), lateral tibial plateau (LTP), medial femoral condyle (MFC), and medial tibial plateau (MTP) was measured. Individual data points presented with mean ± SEM. Data analyzed by two-way ANOVA (95% CI) with Bonferroni post hoc test. Scale bar = 100 μm

We next examined articular cartilage morphology in 11-week-old mutant and control mice using toluidine blue-stained paraffin frontal knee sections (Fig. 3a, b). The average thickness of the calcified articular cartilage and non-calcified articular cartilage in the lateral femoral condyle (LFC), lateral tibial plateau (LTP), medial femoral condyle (MFC), and medial tibial plateau (MTP) from control and *Mig-6*^*over/over*^ male (Fig. 3c, d) and female (Suppl. Fig. 6A-D) mice did not show statistically significant differences. Histological analyses of the knee sections from male and female mice did not show any loss of proteoglycan, fibrillation, or erosion in the articular cartilage of mutant mice.

### Overexpression of Mig-6 in the cartilage induces an osteoarthritis-like phenotype in mice during aging

**Fig. 4:**
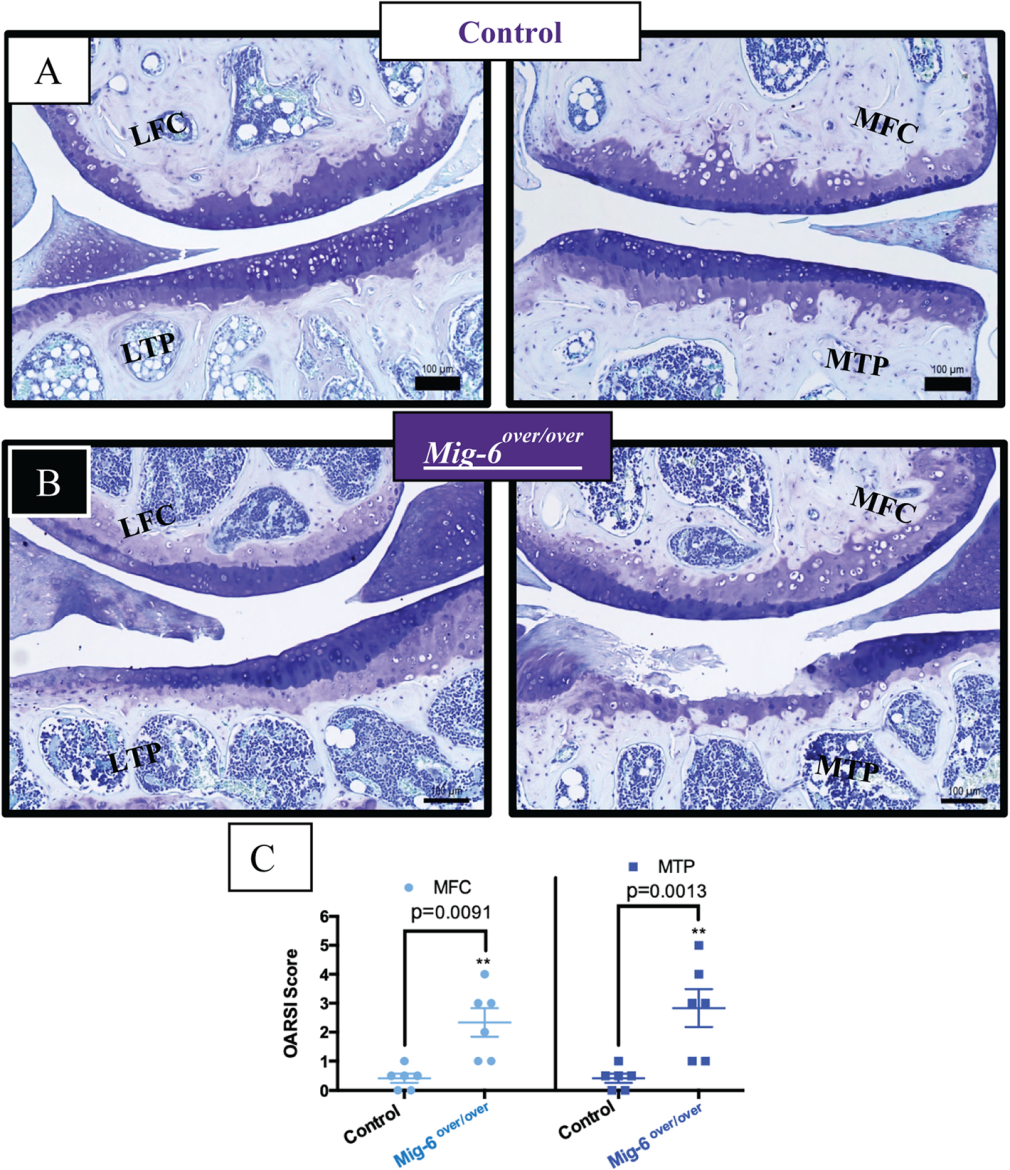
Twelve-month-old *Mig-6*^*over/over*^ male mice develop OA-like cartilage degeneration. Toluidine blue-stained sections of the knee joints from 12-month male control (**a**) and *Mig-6*^*over/over*^ (**b**) mice were evaluated for cartilage damage following the OARSI histopathological scale on two quadrants of the knee: MFC, medial femoral condyle; MTP, medial tibial plateau. OARSI-based cartilage degeneration scores are significantly higher in the MFC and MTP of Mig-6-overexpressing mice, corresponding to the increased damage observed histologically (**c**). Data analyzed by two-way ANOVA with Bonferroni’s multiple comparisons test. Individual data points presented with mean ± SEM. All scale bars= 100 μm. *N* = 9 mice/group

Since aging is a primary risk factor in OA [49], we next examined the knee joints in 12- and 18-month-old control and *Mig-6*^*over/over*^ mice. Toluidine blue-stained sections were evaluated by two blinded observers, using OARSI recommendations [47]. At 12 months of age, male control mice showed minor signs of cartilage damage, such as loss of proteoglycan staining, but no significant structural degeneration (Fig. 4a). However, seven of nine *Mig-6*^*over/over*^ male mice showed more extensive cartilage damage in their medial side (erosion to the calcified layer lesion for 25 to 50% of the medial quadrant) (Fig. 4b). OARSI scoring confirmed increased OA-like damage in mutant mice (Fig. 4c). Similarly, at 18 months of age, the male control group showed minimal cartilage degeneration in 3 of 6 mice (Suppl. Fig. 7A-B). *Mig-6*^*over/over*^ male mice showed more severe cartilage erosion in the medial tibial plateau in 3/6 animals. This result was again supported by significantly increased OARSI cartilage damage scores (Suppl. Fig. 7C). Moreover, for the female group at 12 months, control mice did not show cartilage damage. However, *Mig-6*^*over/over*^ female mice showed signs of OA-like cartilage damage in 3/8 animals (Suppl. Fig. 8A-C). In addition, at 18 months of age, female control mice showed healthy cartilage, and 3/8 *Mig-6*^*over/over*^ female mice showed some proteoglycan loss and cartilage degeneration on the medial side (Suppl. Fig. 9A-C).

### Overexpression of *Mig-6* decreases Sox9 expression

**Fig. 5:**
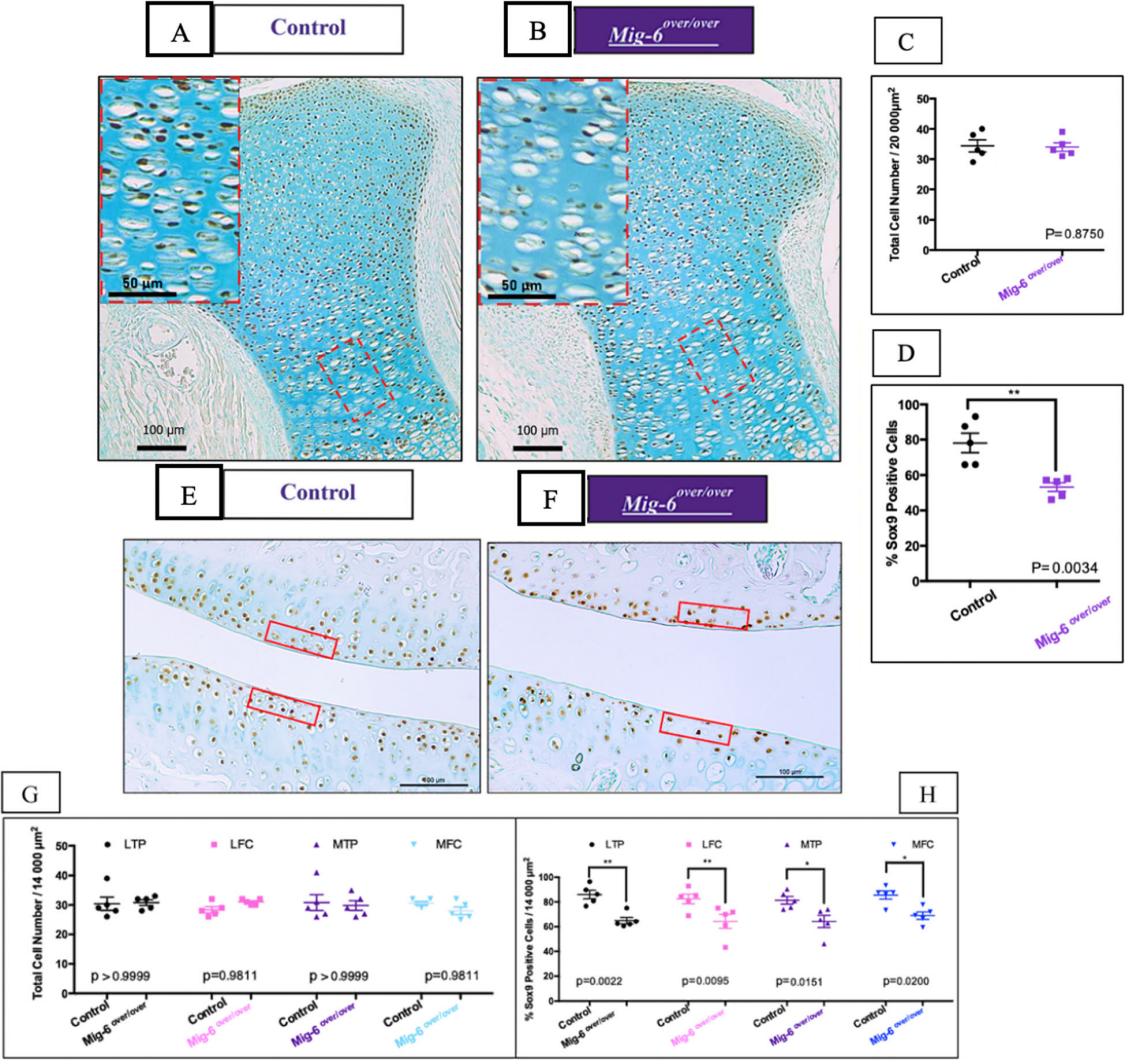
SOX9 immunostaining shows a decrease in positive cells in Mig-6-overexpressing mice at p0 and 6 weeks. Immunostaining of SOX9 in the knee joints of control (**a**/**e**) and *Mig-6*^*over/over*^ (*Mig-6*^*over/over*^*Col2a1-Cre*^*+/−*^*)* (**b**/**f**) mice. Total cell number/area from control and *Mig-6*^*over/over*^ mice (**c**/**g**). Percentage of Sox9-positive cells from control and *Mig-6*^*over/over*^ at p0 and 6 weeks (**d**/**h**). Data analyzed by two-tailed Student *t* tests from 5 mice per group. Individual data points presented with mean ± SEM (*P* < 0.05). Scale bar = 100 μm and 50 μm

During chondrogenesis, the transcription factor SOX9 is required for cartilage formation and normal expression of collagen and aggrecan [50]. The sagittal and frontal sections of paraffin-embedded knees from postnatal day 0 (P0), 6-week-old, 11-week-old, 12-month, and 18-month-old male mice were used for SOX9 immunostaining. At P0, nuclear SOX9 expression was observed in the resting and proliferative zone of the growth plate in both genotypes (Fig. 5a, b). Cell density was not different between genotypes (Fig. 5c). In control mice, 78% of chondrocytes were positive for SOX 9 immunostaining, while the proportion of positive cells was only 53% in *Mig-6*^*over/over*^ mice (Fig. 5d). At 6 weeks old, the total cell number in control male and *Mig-6*^*over/over*^ mice is similar (Fig. 5g), but the percentage of SOX9-positive cells was decreased in mutant mice (Fig. 5h). A similar phenotype was present at 11 weeks (Suppl. Fig. 10A-B). At 12 months of age, SOX9 is present more in the lateral side (LTP and LFC) than the medial side (MTP and MFC) in both strains, with a few positive cells present in the medial side of the control strain. On the other hand, *Mig-6*^*over/over*^ mice showed fewer SOX9-positive cells on the medial side due to the articular cartilage damage (Suppl. Fig. 11A-B). Similar results were found at 18 months of age in *Mig-6*^*over/over*^, with decreased SOX9 immunostaining in their medial side compared to the control (data not shown). For all ages, negative controls did not show staining in chondrocytes.

### Overexpression of Mig-6 decreases expression of lubricin

**Fig. 6:**
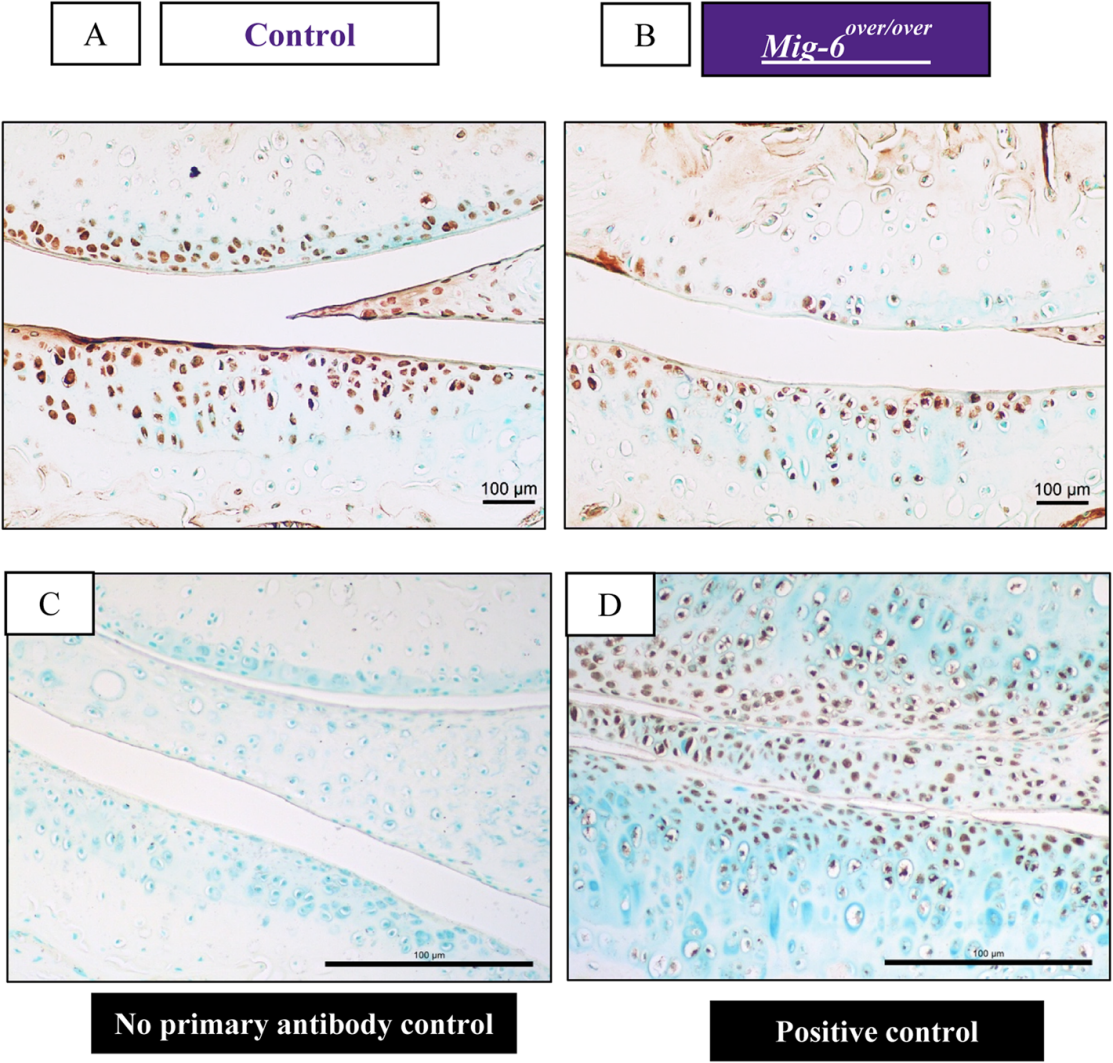
Lubricin immunostaining is slightly decreased in the articular cartilage of cartilage-specific Mig-6-overexpressing mice at 11 weeks of age. Immunostaining of sections of the knee joint indicates the presence of lubricin (PRG4) in superficial zone chondrocytes. IHC reveals no staining for the negative control (**c**). Sections from Mig-6 KO mice served as the positive control (**d**). *N* = 4–5 mice/genotyping. Scale bar = 100 μm

Lubricin (aka PRG4/superficial zone protein) is a proteoglycan that plays an important role as a lubricant in the joint [51]. EGFR signaling is crucial for the cartilage lubrication function and regulates the induction of *Prg4* expression which is necessary for smooth movement [31, 32]. Immunohistochemistry for lubricin in 11-week-old (Fig. 6a–d) and at 12-month-old animals demonstrated less staining in the superficial zone of the medial side of *Mig-6*^*over/over*^ mice than in the control group (Suppl. Fig. 12A-B).

### MMP13 immunostaining in Mig-6-overexpressing and control mice

Matrix metalloproteinase (MMP13) is highly expressed in OA [52, 53]. The frontal sections of the knees from 12- and 18-month-old control and *Mig-6*^*over/over*^ male mice were used for MMP13 immunohistochemistry. At 12 months, pericellular staining was observed in the lateral articular cartilage of male mice from both genotypes, along with the expected subchondral bone staining (Suppl. Fig. 13A-B). Less staining was observed on the medial side of control mice while advanced cartilage degeneration in mutant mice precluded staining. Negative controls did not show staining in the cartilage or subchondral bone. The articular cartilage from 18-month-old mice showed similar staining patterns and intensity of MMP13 immunostaining in the lateral side of both genotypes; however, in the medial side of *Mig-6*^*over/over*^ mice, MMP13 staining is seen on the cartilage surface (lesion sites) and also observed in the subchondral bone (data not shown).

## Discussion

The maintenance of articular cartilage homeostasis relies on a dynamic equilibrium involving growth factors [54], genetics [55], mechanical forces [56], obesity, and injury that all play a role in the onset of osteoarthritis [57]. A better understanding of the underlying molecular mechanism is required to design therapies for preventing the progression of OA. Recent studies from our laboratory and others have identified the epidermal growth factor receptor (EGFR) and Mig-6 as possible mediators of articular cartilage homeostasis [31, 33, 34, 40]. *Mig-6* is a cytosolic protein and negative feedback regulator of EGFR signaling [58]; thus, *Mig-6* can be a potential tumor suppressor [41, 59–62]. In addition, whole-body knockout of the *Mig-6* gene in mice results in degenerative joint disease [37]. We also have shown previously that constitutive cartilage-specific deletion of *Mig-6* (Mig-6 KO) results in increased articular cartilage thickness and cell density in the joints of 12-week-old mice [40]. Cartilage-specific Mig-6 KO mice show the same anabolic effect in the joint cartilage at 21 months of age (unpublished).

Previous research demonstrates that Mig-6 overexpression acts as a negative feedback regulator of EGFR-ERK signaling [41]; however, these studies did not yet analyze joint tissues. Since our studies suggest dosage- and/or context-specific roles of EGFR signaling in joint homeostasis and OA [34], we now examined whether overexpression of Mig-6 alters these processes. Here, we report that cartilage-specific constitutive overexpression of *Mig-6* did not cause cartilage degeneration in young mice, but early-onset OA in middle-aged mice. While we observed some effects of Mig-6 overexpression on bone length and weight, these effects were subtle and not accompanied by major morphological or histological changes in the growth plate cartilage, overall skeletal morphology, or body composition. A previous study showed that the deletion of EGFR in bone tissue (*Col1-Cre Egfr*^*Wa5/f*^) resulted in shorter femurs compared to wild-type mice [63], consistent with our findings. The EGFR network is essential during long bone development, since previous studies have shown that EGFR- or TGFα-deficient mice exhibit a widened zone of hypertrophic chondrocytes [23, 64]. Moreover, Qin and colleagues have shown that the administration of the EGFR inhibitor, gefitinib, into 1-month-old rats results in an enlarged hypertrophic zone due downregulation of MMP-9, MMP-13, and MMP-14 [30]. Together, these data suggest a critical role of EGFR during endochondral ossification and elucidate the downstream mechanism of EGFR [65]. Further research is required to provide more evidence of EGFR/*Mig-6*^*over/over*^ signaling during bone formation, but many of these effects are relatively subtle and transient, and likely unrelated to the much more severe phenotypes observed later. Histologically, our findings showed that mice with cartilage-specific *Mig-6* overexpression showed healthy articular cartilage with no significant difference in articular cartilage thickness from the control group at the ages of 6 weeks and 11 weeks. However, *Mig-6*^*over/over*^ mice developed severe degeneration of the articular cartilage with aging. More prevalent, the knee joints of *Mig-6*^*over/over*^ male mice showed significantly advanced cartilage degeneration. The same pattern, but with more severe damage, was seen in 18-month-old mice. As previously described, sex hormones play a role in OA disease where male mice develop more severe OA [66]. SOX9 is crucial in chondrogenesis during endochondral bone formation, articular cartilage development, and cartilage homeostasis [50]. Previous in vivo models using cartilage (Col2)-Cre- or limb mesenchyme (Prx1)-Cre*-*specific ablation of *Mig-6* showed increased expression of SOX9 in the articular cartilage. Consistent with these data, we show that the percentage of SOX9-positive chondrocytes was decreased in the knee joints of 6- and 11-week-old male *Mig-6*^*over/over*^ mice compared to controls, despite the absence of histological defects in articular cartilage. The percentage of SOX9-expressing cells was also reduced in *Mig-6*^*over/over*^ mice at later ages. However, our previous in vitro studies suggested that TGFα suppresses the expression of anabolic genes such as Sox9, type II collagen, and aggrecan in primary chondrocytes [21]. A potential explanation for these conflicting results is the context-specific role of EGFR signaling in the cartilage, as discussed previously [34]; this pathway can both promote cartilage degeneration and confer protection of the cartilage, depending on the context. Another potential explanation is the difference between in vivo and in vitro experiments. Our data from the current study suggest that reduction in Sox9 expression precedes the degeneration of articular chondrocytes in our mutant mice. In addition, we observed decreased expression of lubricin/PRG4 in these joints, which might also contribute to the observed joint pathologies. PRG4 has been shown to be regulated by EGFR signaling before [31, 32], in support of our findings. While the EGFR is the best characterized substrate of Mig-6, other substrates have been described. Mig-6 binds to different proteins such as the cell division control protein 42 homolog (Cdc42) [67], proto-oncoprotein (c-Abl) [68], and the hepatocyte growth factor receptor (c-Met) [69]. While we cannot exclude that deregulation of these other substrates contributes to the observed phenotypes, the similarities of defects in our mice with those seen upon cartilage-specific deletion of EGFR suggest that decreased EGFR signaling is the main cause for the advanced OA observed in our mutant mice. Nevertheless, it will be important to determine whether signaling through cMet and other pathways is altered as well.

Future studies will include an examination of Mig-6-overexpressing mice in other models of OA, such as the post-traumatic model of DMM (destabilization of the medial meniscus) surgery. As discussed above, the role of the EGFR/Mig-6 pathway in OA is context-specific. Thus, we cannot extrapolate from our aging model to all forms of OA. In addition, the role of Mig-6 in human OA will require further studies. While there is plenty of evidence for crucial roles of the EGFR pathway in human OA, and Mig-6 is a crucial regulator of this pathway, more direct analyses of this role in human cartilage are required [21, 70, 71].

Mig-6 expression is heavily regulated. While its expression in human OA has not been studied thoroughly, one can easily imagine upregulation by a number of biochemical (such as EGFR and other growth factors, as in other tissues) or biomechanical factors. Mig-6 overexpression has been demonstrated in OA cartilage in dogs [38], suggesting that the same could occur in human OA. More generally, the EGFR pathway, through which Mig-6 primarily acts, has been shown to be deregulated in human OA in several studies [21, 34, 71]. Thus, the current study is highly relevant to the pathogenesis of human OA.

## Conclusion

In conclusion, we show for the first time that cartilage-specific Mig-6 overexpression in mice results in reduced EGFR activity in chondrocytes, reduced SOX9 and PRG4 expression, and accelerated development of OA. These data highlight the important and context-specific role of the EGFR-Mig-6 signaling pathway in joint homeostasis and point towards potential targeting of this pathway for OA therapy.

## Supplementary information

**Additional file 1.** Supplementary figures.

## Data Availability

The dataset of the current manuscript is available upon request from the corresponding author.

## Abbreviations

*Acan*: Aggrecan gene
BMD: Bone mineral density
c-Abl: Non-receptor tyrosine kinase proto-oncoprotein
CAGGS: Chicken beta actin-cytomegalovirus hybrid
Cdc42: Cell division control protein 42
cDNA: Complementary DNA
c-Met: Hepatocyte growth factor receptor
*Col2a1*: Type II collagen gene
ECM: Extracellular matrix
EGFR: Epidermal growth factor receptor
ERK1/2: Extracellular signal-regulated kinase 1 and 2
ERRFI1: ErbB receptor feedback inhibitor 1
*Gadph*: Glyceraldehyde 3-phosphate dehydrogenase gene
GE: General electric
HRP: Horseradish peroxidase
IGF-1: Insulin growth factor 1
KO: Knockout
LFC: Lateral femoral condyle
LSL: “Stop Cassette” flanked by LoxP (LoxP-Stop Cassette-LoxP)
LTP: Lateral tibial plateau
Lubricin: PRG4/superficial zone protein
MFC: Medial femoral condyle
*Mig-6*: Mitogen-inducible gene 6; RALT; Gene 33
MMP13: Matrix metalloproteinase 13
OA: Osteoarthritis
OARSI: Osteoarthritis Research Society International
P0: Newborn mouse (postnatal day 0)
PBS: Phosphate-buffered solution
pEGFR: Phosphorylated epidermal growth factor receptor
PI3K: Phosphoinositide 3-kinase
ROI: Region of interest
SB3: Cortical bone-mimicking calibrator
SOX9: Sex-determining-region-Y box 9
TGFα: Transforming growth factor alpha
μCT: Micro-computerized tomography
