# Supplementary material for: Overexpression of MIG-6 in the cartilage induces an osteoarthritis-like phenotype in mice
Source: Arthritis Res Ther. 2020 May 19;22:119. doi: 10.1186/s13075-020-02213-z (PMC7236969; doi:10.1186/s13075-020-02213-z)
Supplement: Supplementary file 1 — Additional file 1. Supplementary figures. [file 13075_2020_2213_MOESM1_ESM.pdf]

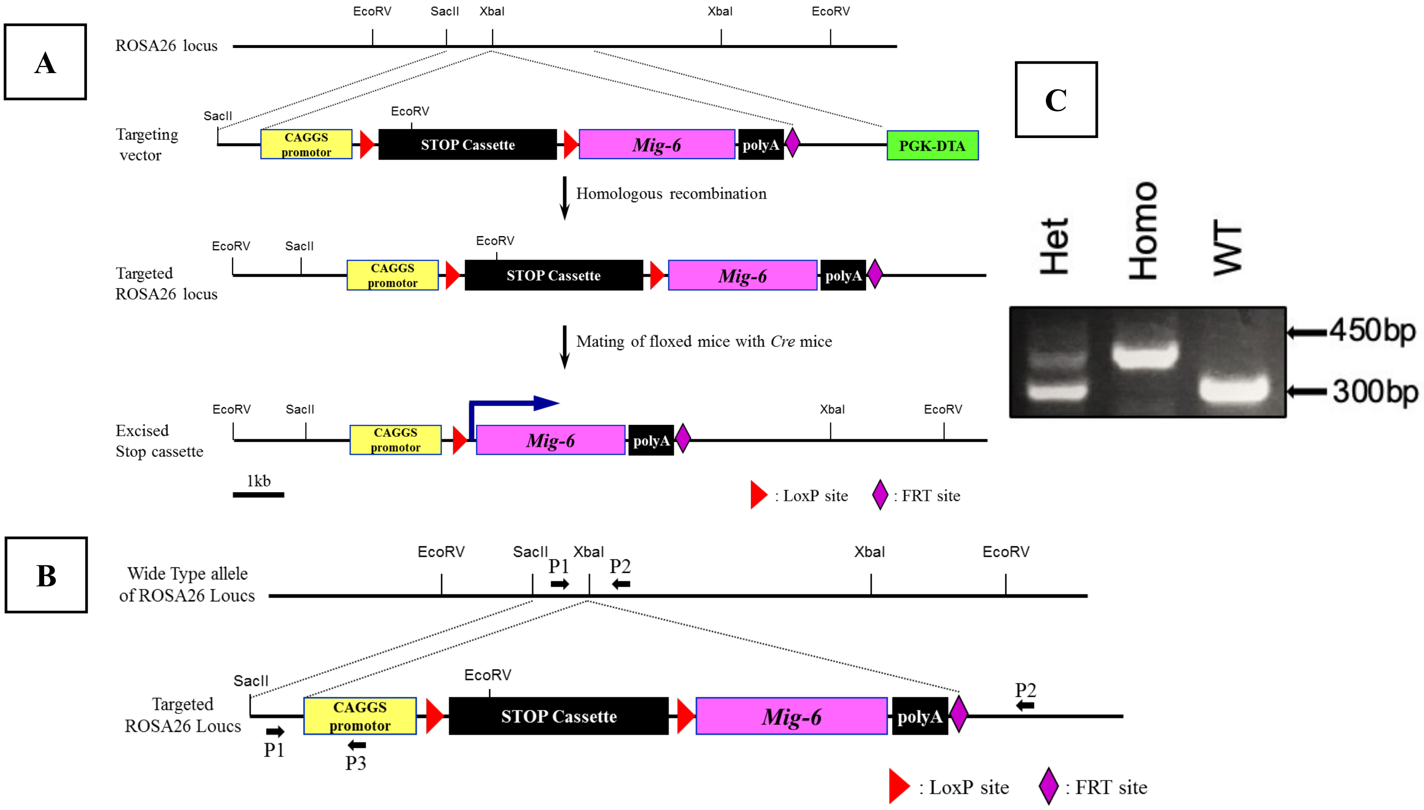

D

PCR Primer	5' to 3'
P1	GGG GGA GGG GAG TGT TGC
P2	CCA TTT TCC TTA TTT GCC CCT ATT
P3	GGG CCA TTT ACC GTC ATT

E

RT- PCR Primer	*Mig-6* $^{over/over}$
Forward	GGGGTCCCGCTCCCTT
Reverse	ACATTGTGCCCCACTCTGG

Supplementary Figure 1) Construction of the targeting vectors and generation of *Mig-6*$^{over/over}$ mice. Adapted from Kim, T. H. *et al.* Mig-6 suppresses endometrial cancer associated with pten deficiency and ERK activation. *Cancer Res.* **74,** 7371–7382 (2014). **(A)** The overexpression of *Mig-6* is accomplished by placing the transcription of *Mig-6* under the control of a ubiquitously expressed promoter, the chicken b actin-cytomegalovirus hybrid (CAGGS) promoter. The construction also contained the "Stop Cassette" flanked by LoxP sites (LSL). **(B)** PCR strategy. P1 and P2 can amplify a 300 bp fragment from the wild-type allele, whereas P1 and P3 can amplify a 450 bp fragment from the targeted ROSA26 locus allele. **(C)** A representative agarose gel image of PCR genotyping, heterozygous (Het), wild-type (wt) and homozygous (Homo). **(D)** PCR primer sequence for wild type and *Mig-6*^{LSL} allele. **(E)** RT- PCR primer sequence for *Mig-6*$^{over/over}$.

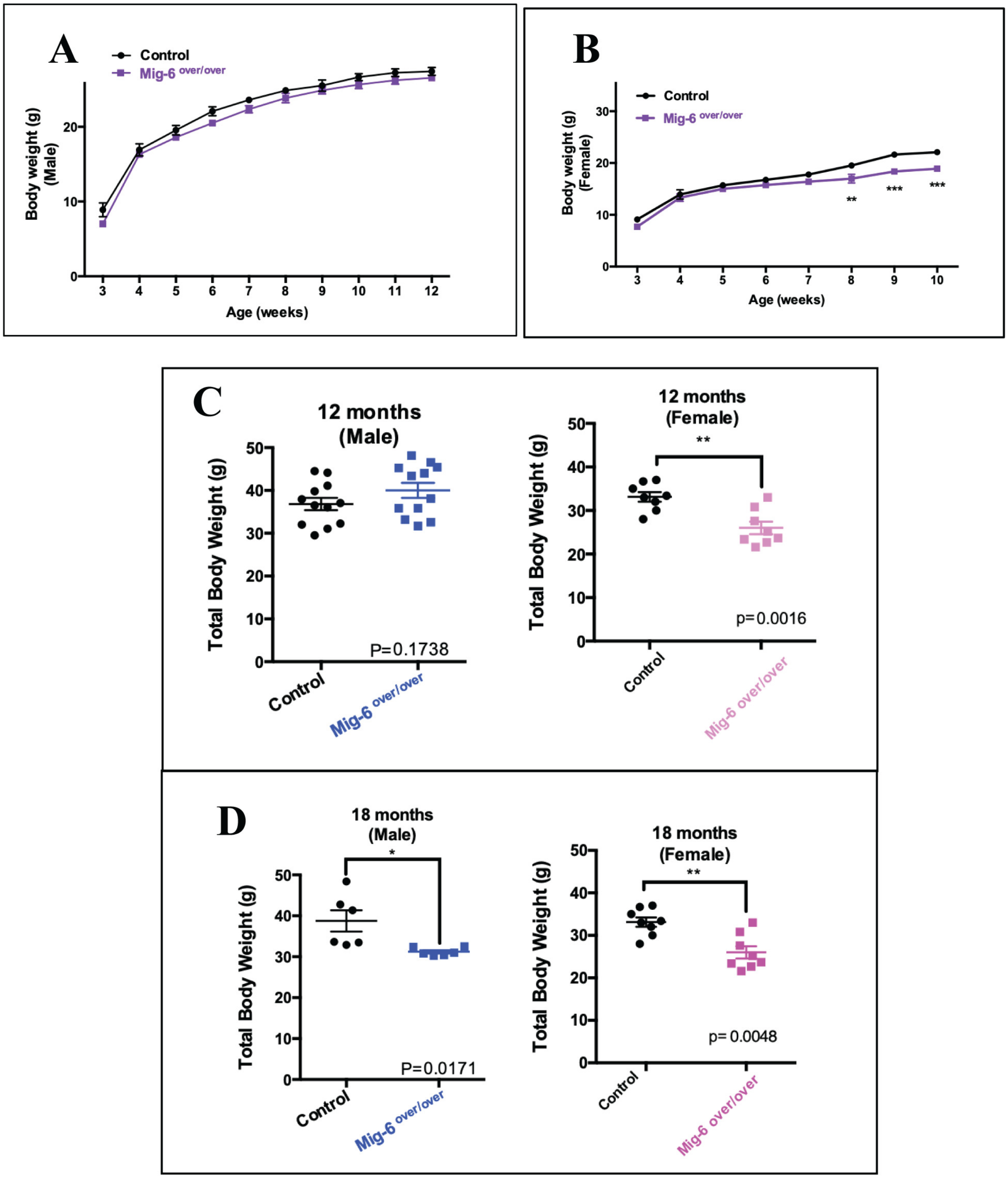

Supplementary Figure 2) Body weight of control and *Mig-6$^{over/over}$* male and female mice during growth. Body weight of male Mig-6 overexpression mice did not show any significant differences compared to control **(A)** Female Mig-6 overexpression mice showed statistically significant differences compared to control at 8w, 9w and 10w **(B).** Two-Way ANOVA was used with Bonferroni post hoc analysis (n=5/genotyping). Data are presented with mean and error ± SEM ($P<0.05$). Weights of 12 months old **(C)** and 18 months old **(D)** male and female cartilage specific *Mig-6$^{over/over}$* mice and controls taken immediately prior to sacrifice. Individual data points presented with mean ± SEM ($P<0.05$). Data analyzed by two tailed student t-tests from 6-12 mice per group (age/genotyping).

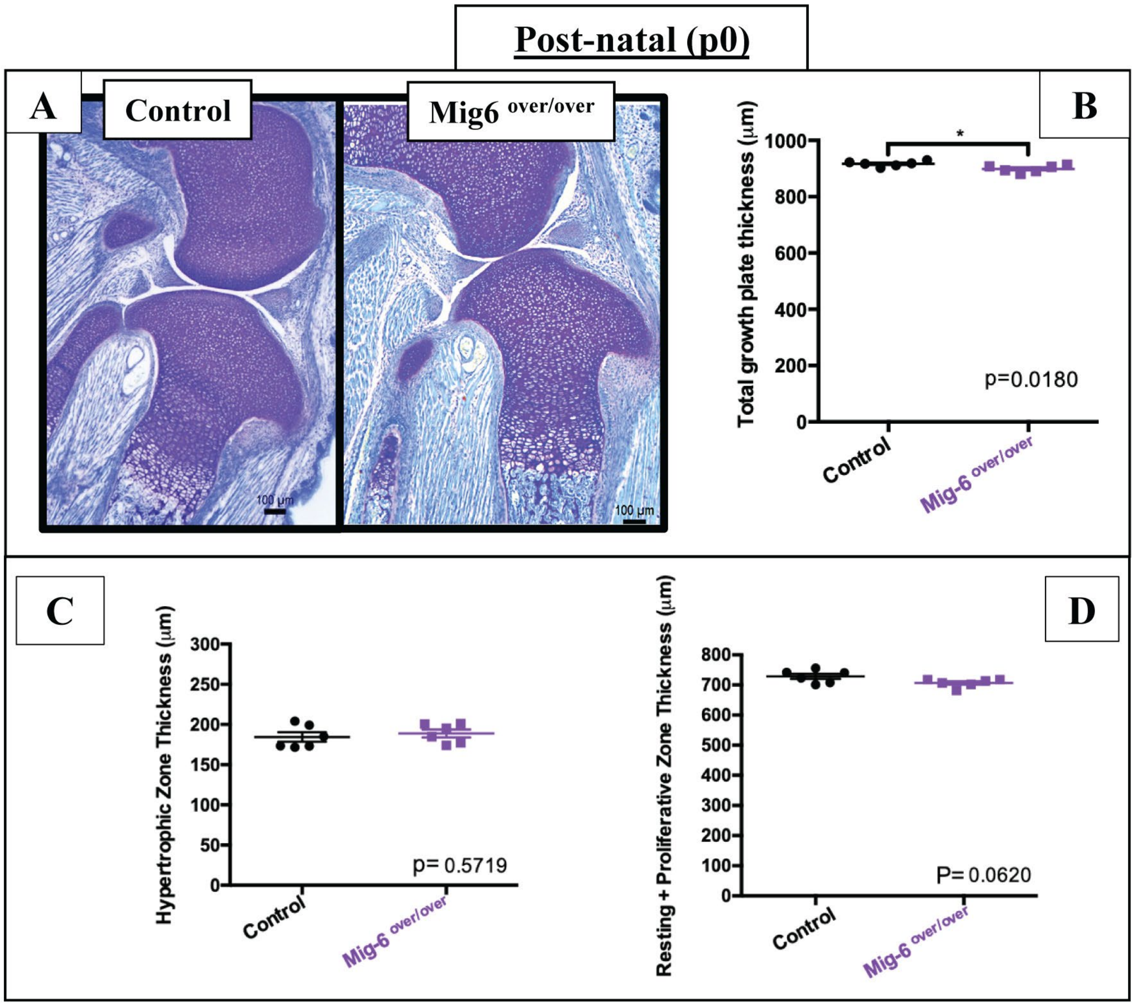

Supplementary Figure 3) Cartilage-specific Mig6-overexpressing mice display no major developmental phenotype. Representative toluidine Blue staining on postnatal day 0 (P0) of *Mig-6*$^{over/over}$ **(A)** and control animals. Thickness of total proximal tibia growth plates in the mice containing articular cartilage specific mitogen inducible gene 6 overexpression (n=6), when compared to age matched controls (n=6) was significantly decreased when analyzed by two tailed student t-tests **(B)**; The average of hypertrophic zone thickness from postnatal day 0 in the mice containing articular cartilage specific mitogen inducible gene 6 overexpression had mean of 188.9 µm and control mice had mean of 184.4 µm **(C).** The thickness of the combined resting and proliferative zones from the control had mean of 728 µm and *Mig-6*$^{over/over}$ 706.5 µm *Mig-6*$^{over/over}$**(D).** Therefore, there was no significant differences within the groups. Individual data points presented with mean ± SEM analyzed by two tailed student t-tests; ($P<0.05$).

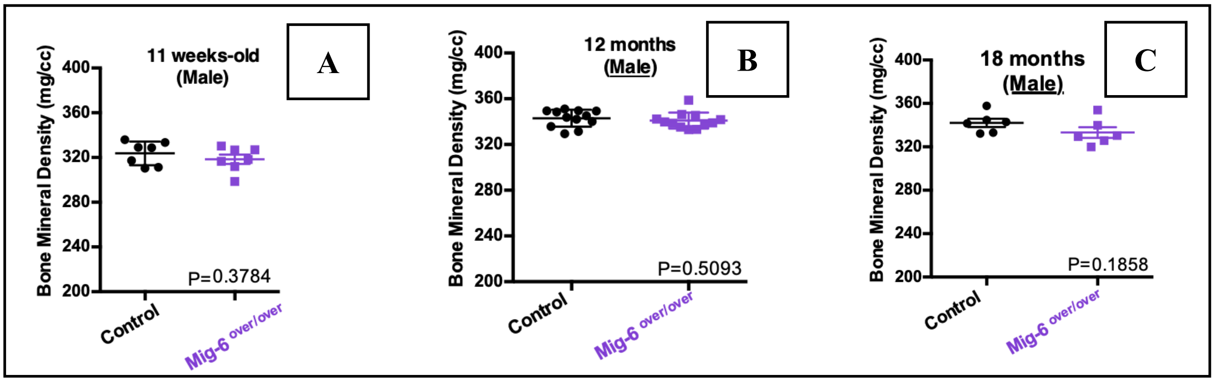

Supplementary Figure 4) Bone mineral densities were measured from µCT scan volumes from control and *Mig-6$^{over/over}$* male mice. (A) Mean bone mineral densities were 323.7 mg/cc (control) and 318.5 mg/cc (*Mig-6$^{over/over}$*) at 11 weeks-old. Moreover, **(B)** At 12 months of age male *Mig-6$^{over/over}$* mice had mean bone mineral density (342.9 mg/cc) and controls male mice (341.0 mg/cc). **(C)** At 18 months of age, there were no significance difference between the mean bone mineral density from control male mice (342.1 mg/cc) and male *Mig-6$^{over/over}$* (333.2 mg/cc). There were not significantly different among 11 weeks-old, 12 and 18 months for bone mineral density for male control and Mig-6 overexpression mice. Individual data points presented with mean ± SEM ($P<0.05$). Data analyzed by two tailed student t-tests from 6-12 mice per group (age/gender).

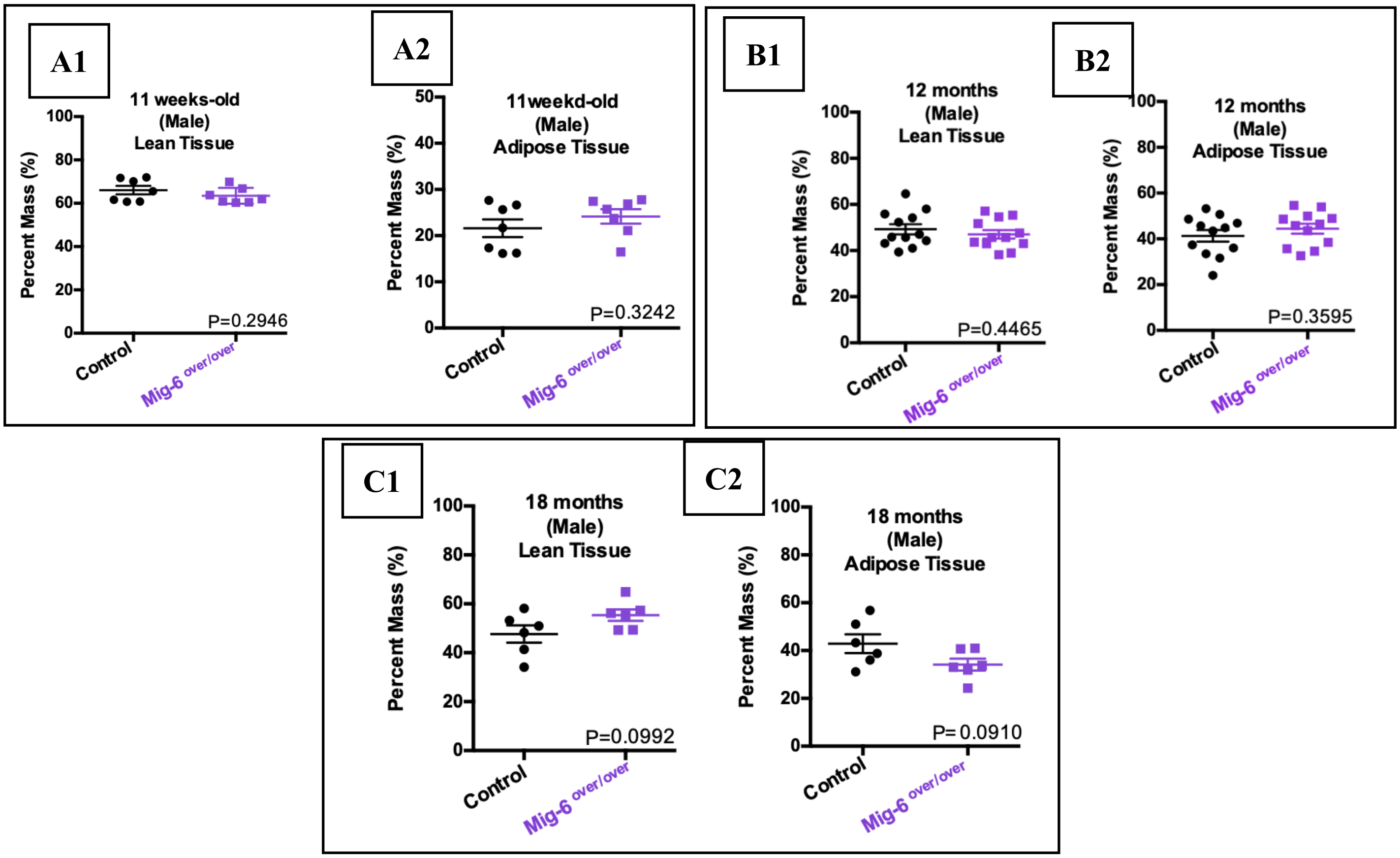

Supplementary Figure 5) Body composition and body mass from growth and aging male control and *Mig-6*$^{over/over}$. Body composition was calculated from control and *Mig-6*$^{over/over}$ male mice. At 11 weeks-old **(A1/A2)**, 12 months **(B1/B2)** and 18 months **(C1/C2)** neither the average lean mass percent or mean body fat were not statistically significant between genotypes. Individual data points presented with mean ± SEM ($P<0.05$). Data analyzed by two tailed student t-tests from 6-12 mice per group (age/genotyping).

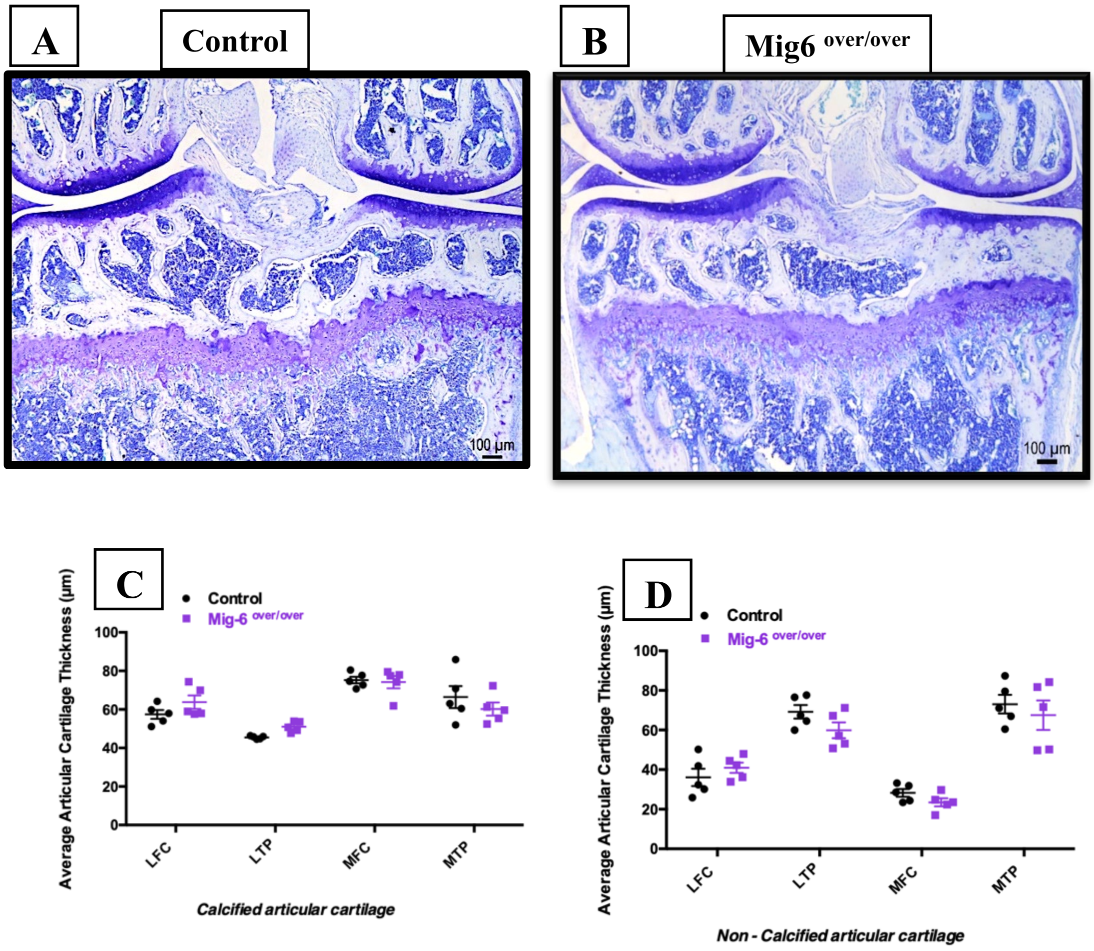

Supplementary Figure 6) Articular cartilage from 11 weeks-old *Mig-6*$^{over/over}$ female mice appeared healthy during skeletal maturity. Representative (n=5/group, toluidine blue) stained frontal sections of knee joints from 11-week-old control **(A)** and Mig-6over **(B).** Mig-6 overexpressors mice show similar articular cartilage thickness when compared to controls at 11 weeks-old female mice. The average thickness of the calcified articular cartilage **(C)** and non-calcified articular cartilage **(D)** in the lateral femoral condyle (LFC), lateral tibial plateau (LTP), medial femoral condyle (MFC), medial tibial plateau (MTP) was measured. Individual data points presented with mean ± SEM. Data analyzed by two-way ANOVA (95% CI) with Bonferroni post-hoc test. Scale bar = 100µm.

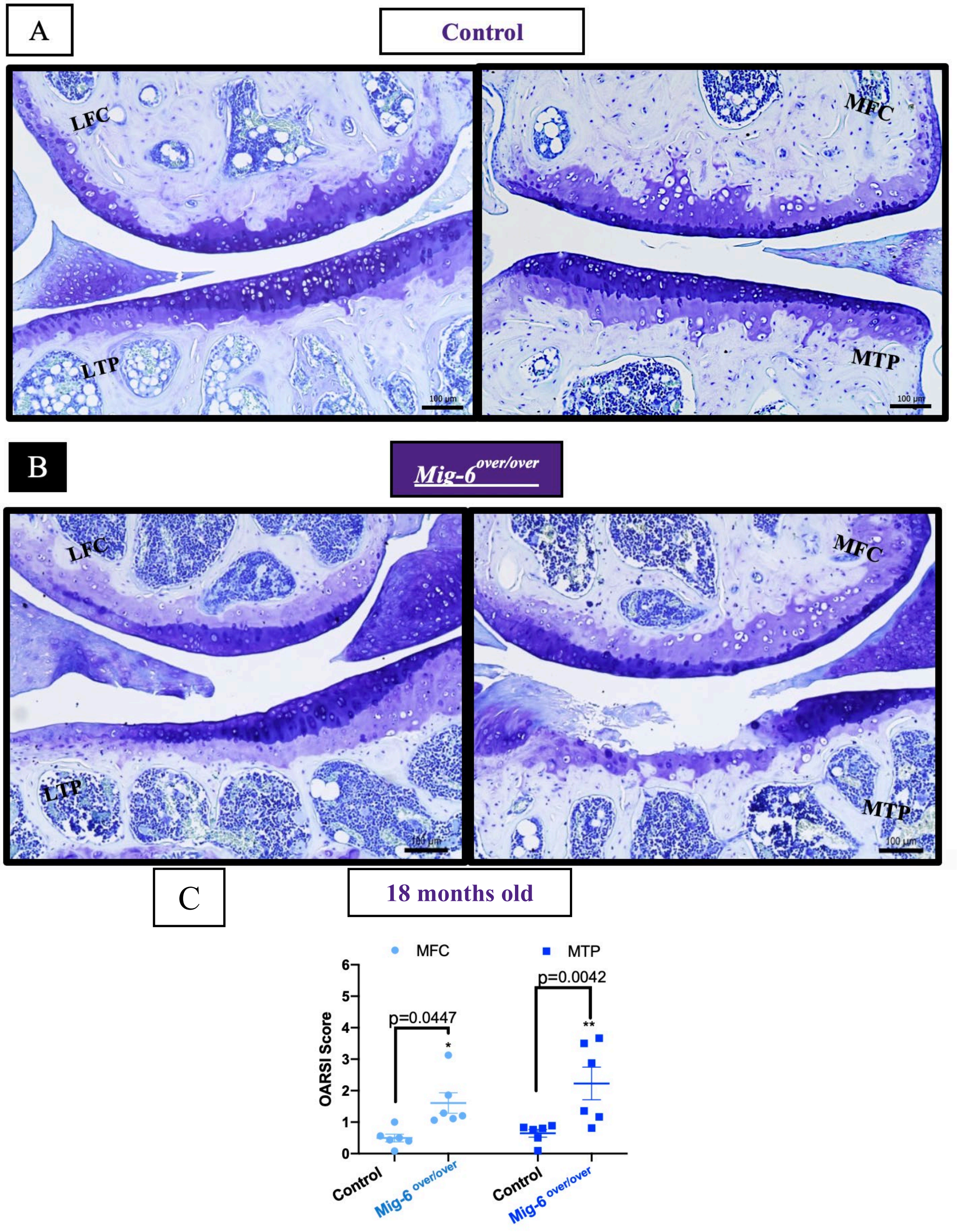

Supplementary Figure 7) 18 months old *Mig-6$^{over/over}$* mice leads to advanced OA-like cartilage. Representative images of Toluidine Blue stained sections of knee joints from 18-month male control **(A)** and male Mig-6 over **(B)** mice were evaluated for cartilage damage following OARSI histopathological scale on the two quadrants of the knee: MFC = medial femoral condyle, MTP = medial tibial plateau. OARSI based cartilage degeneration scores are higher both in the MFC and MTP of Mig-6 overexpressing mice, corresponding to the increased damage observed histologically. **(C)** Data analyzed by two-way ANOVA with Bonferroni's multiple comparisons test. Individual data points presented with mean ± SEM. All scale bars =100 μm. N = 6 mice/group.

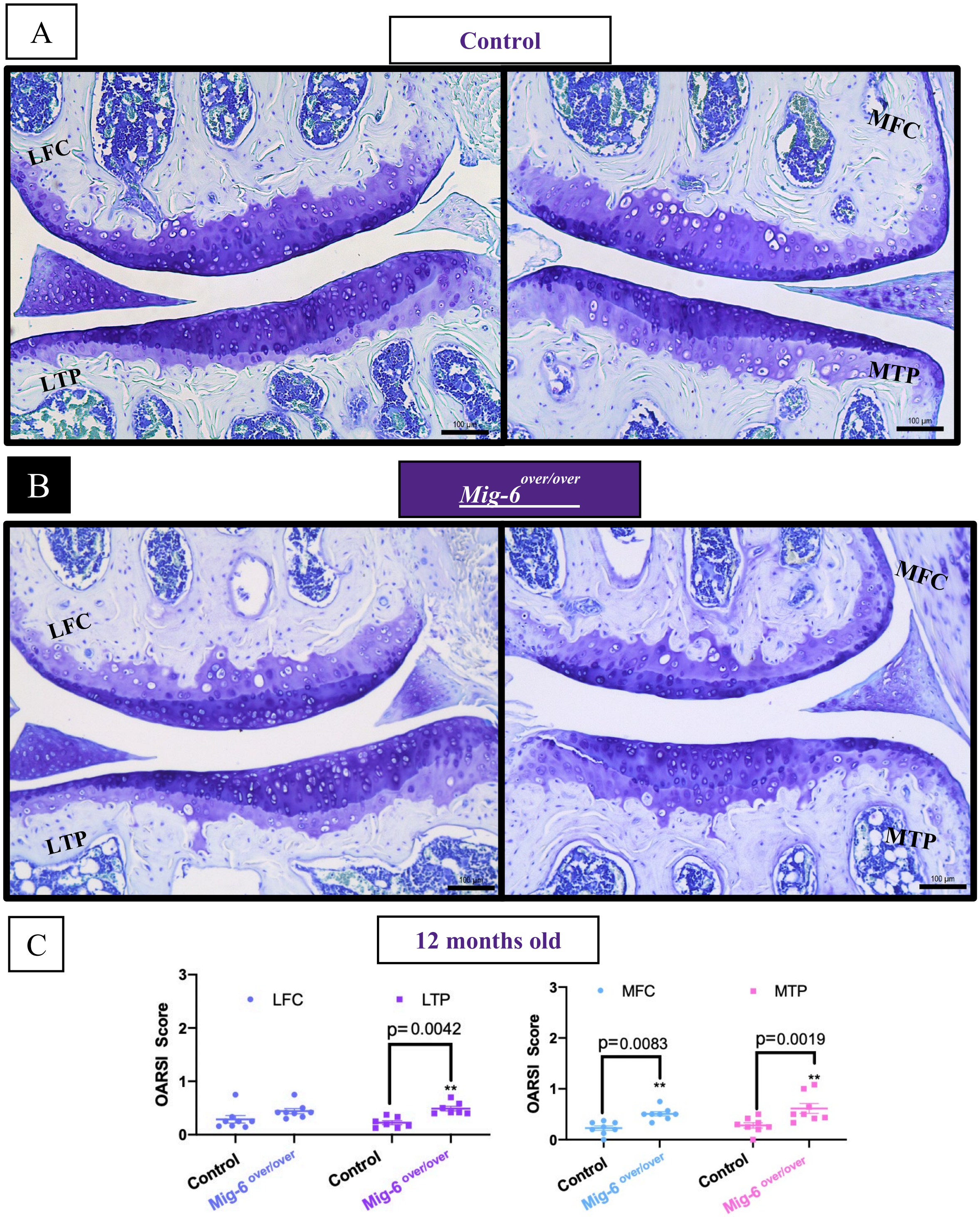

Supplementary Figure 8) 12 months old *Mig-6*$^{over/over}$ female mice showed little damage. Representative images of Toluidine Blue stained sections of knee joints from 12-month female control **(A)** and female Mig-6 over **(B)** mice were evaluated for cartilage damage following OARSI histopathological scale on the four quadrants of the knee: LFC = lateral femoral condyle, LTP = lateral tibial plateau, MFC = medial femoral condyle and MTP = medial tibial plateau. OARSI based cartilage degeneration scores are higher both in the MFC and MTP of Mig-6 overexpressing mice, corresponding to the increased damage observed histologically **(C)**. Data analyzed by two-way ANOVA with Bonferroni's multiple comparisons test. Individual data points presented with mean ± SEM. All scale bars =100 µm. N = 8 mice/group. Scale bar = 100µm.

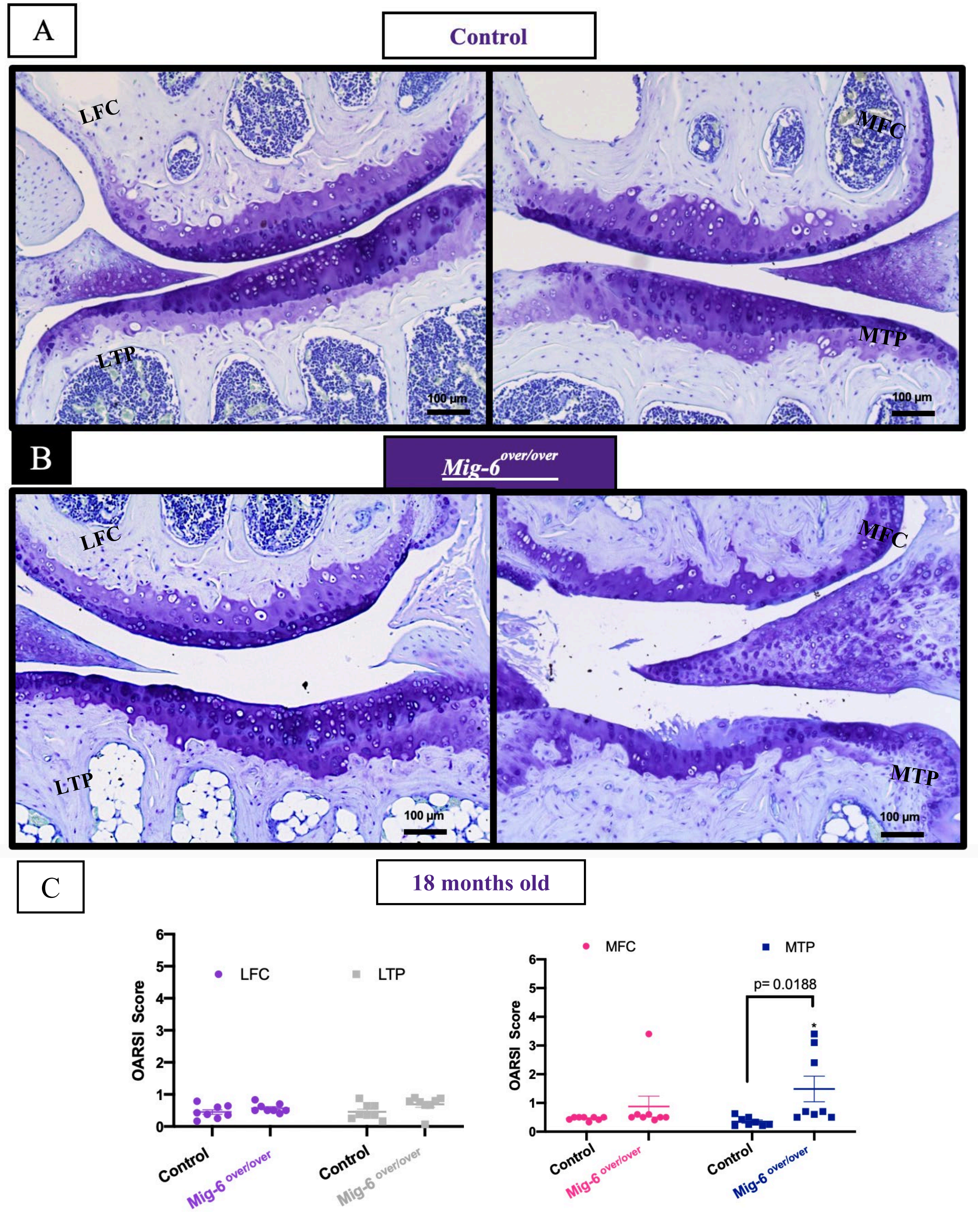

Supplementary Figure 9) 18 months old *Mig-6*$^{over/over}$ female mice showed little damage. Representative images of Toluidine Blue stained sections of knee joints from 18-month female control **(A)** and female Mig-6 over **(B)** mice were evaluated for cartilage damage following OARSI histopathological scale on the four quadrants of the knee: LFC = lateral femoral condyle, LTP = lateral tibial plateau, MFC = medial femoral condyle and MTP = medial tibial plateau. OARSI based cartilage degeneration scores are higher both in the MFC and MTP of Mig-6 overexpressing mice, corresponding to the increased damage observed histologically. **(C)**. Data analyzed by two-way ANOVA with Bonferroni's multiple comparisons test. Individual data points presented with mean ± SEM. All scale bars =100 μm. N = 8 mice/group. Scale bar = 100μm.

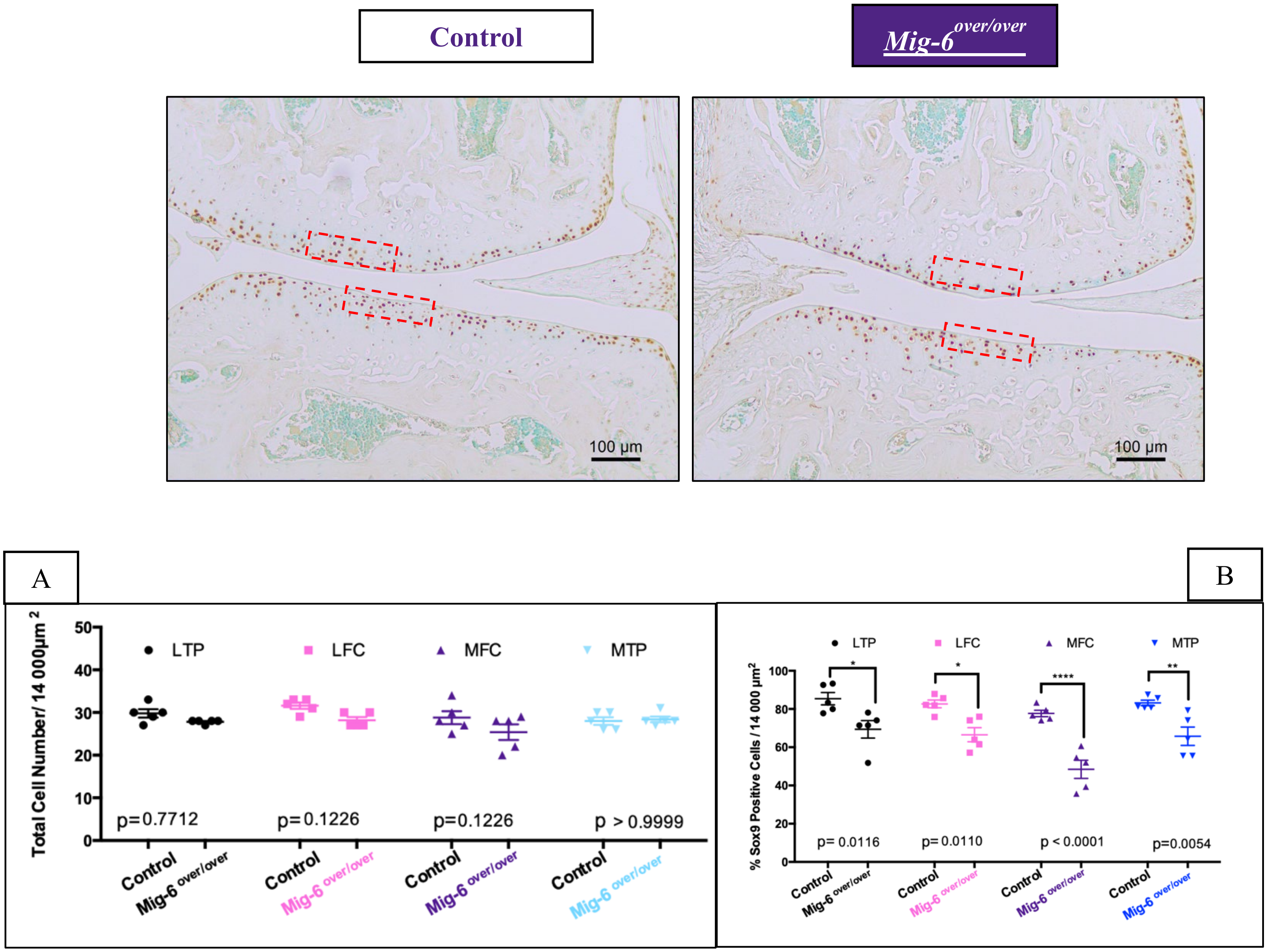

Supplementary Figure 10) SOX9 immunostaining shows a decrease in Mig-6 overexpressors mice at 11 weeks-old. Ratio between the total cell number from control and Mig-6over at 11 weeks-old male mice **(A).** Ratio between the percentage of Sox9 positive cells from control and Mig-6over at 11 weeks-old male mice **(B).** Data analyzed by two-way ANOVA (95% CI) with Bonferroni post-hoc test. Individual data points presented with mean ± SEM; N= 5 mice/genotyping. LFC = lateral femoral condyle, LTP = lateral tibial plateau, MFC = medial femoral condyle and MTP = medial tibial plateau. Scale bar = 100µm.

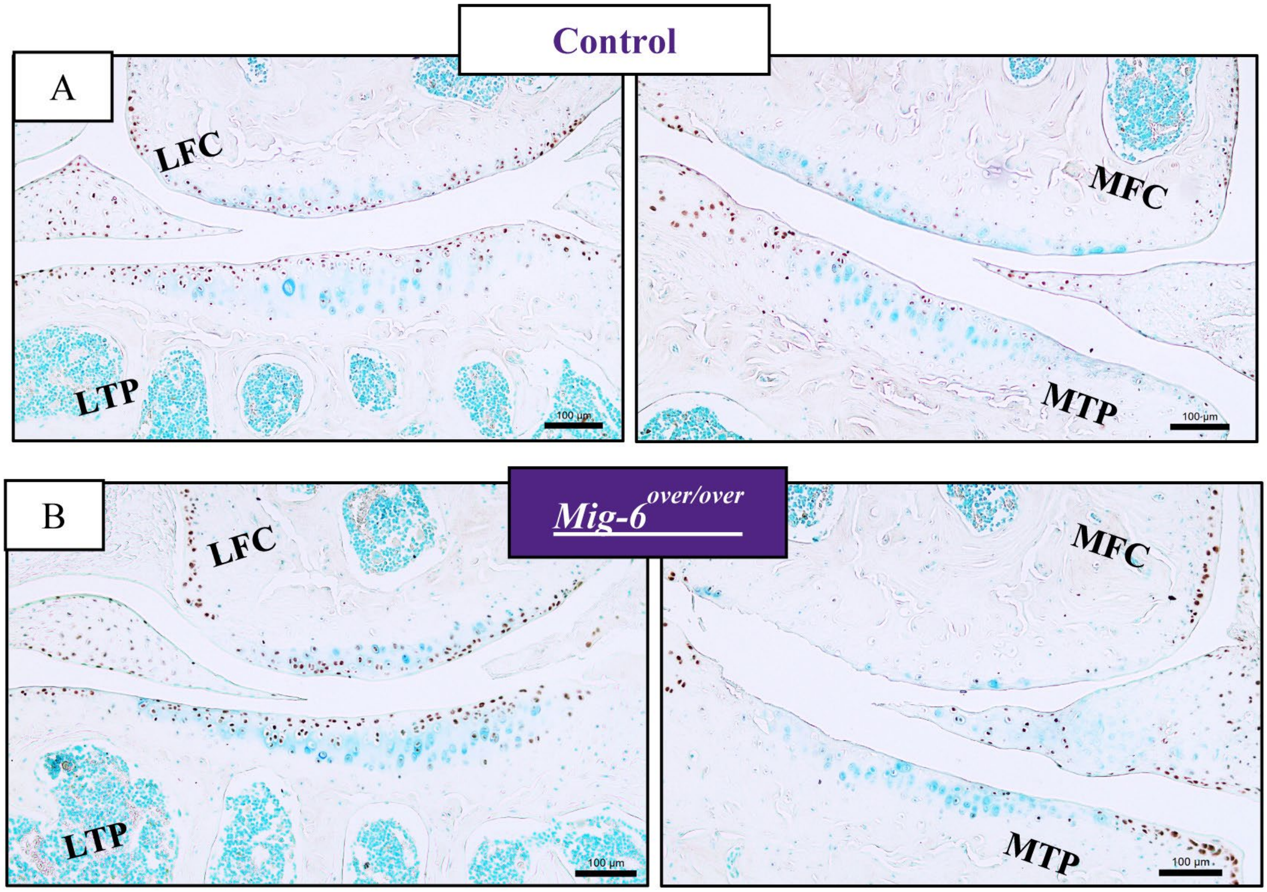

Supplementary Figure 11) 12-month-old cartilage specific Mig-6 overexpressing mice show decreased SOX9 immunostaining. Representative SOX9 immunostained in male mice (n=5) in MFC and MTP show decreased staining intensity in Mig-6 over mice **(B)** when compared to control **(A).** No primary control for articular cartilage **(C).** LFC = lateral femoral condyle, LTP = lateral tibial plateau, MFC = medial femoral condyle and MTP = medial tibial plateau. Scale bar = 100µm.

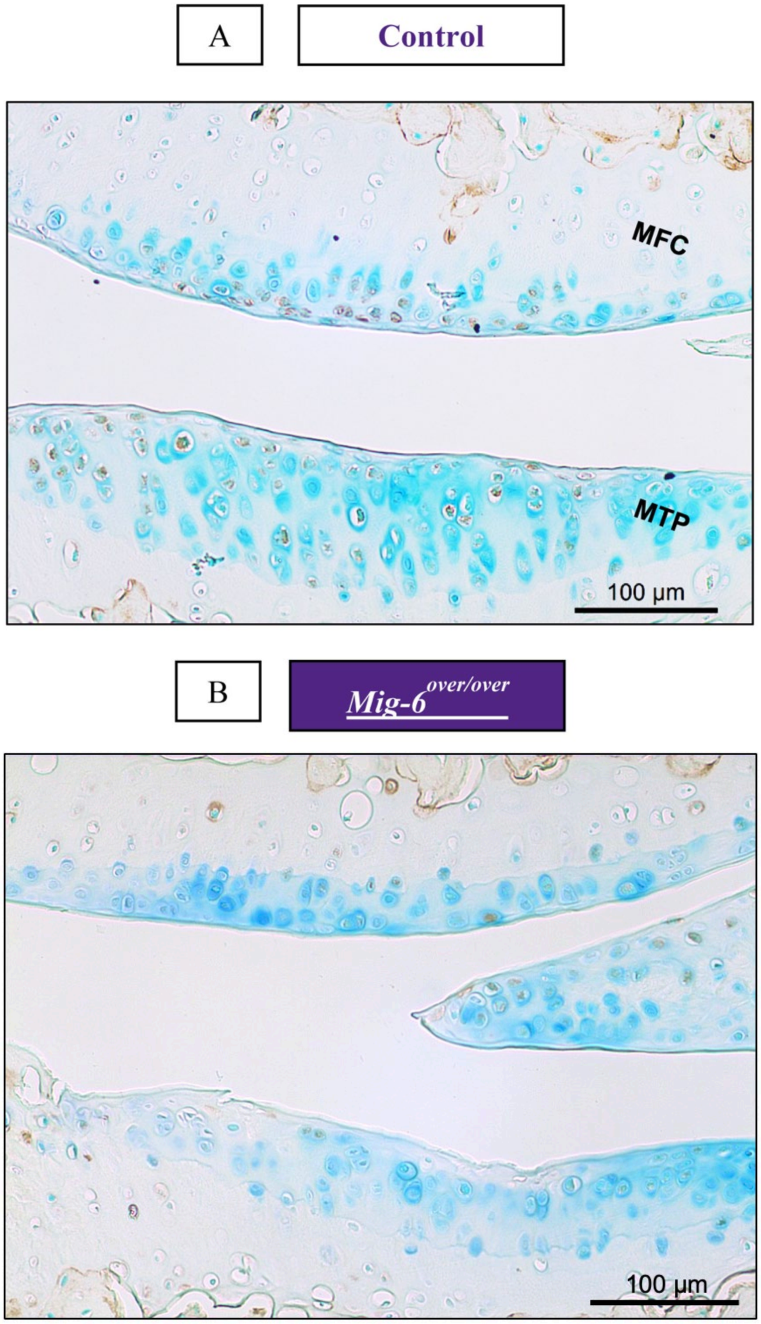

Supplementary Figure 12) Lubricin immunostaining is decreased in the articular cartilage of cartilage specific Mig-6 overexpressing mice at 12 months of age. Immunostaining of sections of the knee joint indicate the presence of Lubricin (*PRG4*) in the superficial zone chondrocytes. Representative Lubricin immunostained in male mice (n=5) in MFC and MTP show decreased staining intensity in Mig-6 over mice **(B)** when compared to control **(A).** N=4-5 mice/genotyping. MFC = medial femoral condyle and MTP = medial tibial plateau. Scale bar = 100µm.

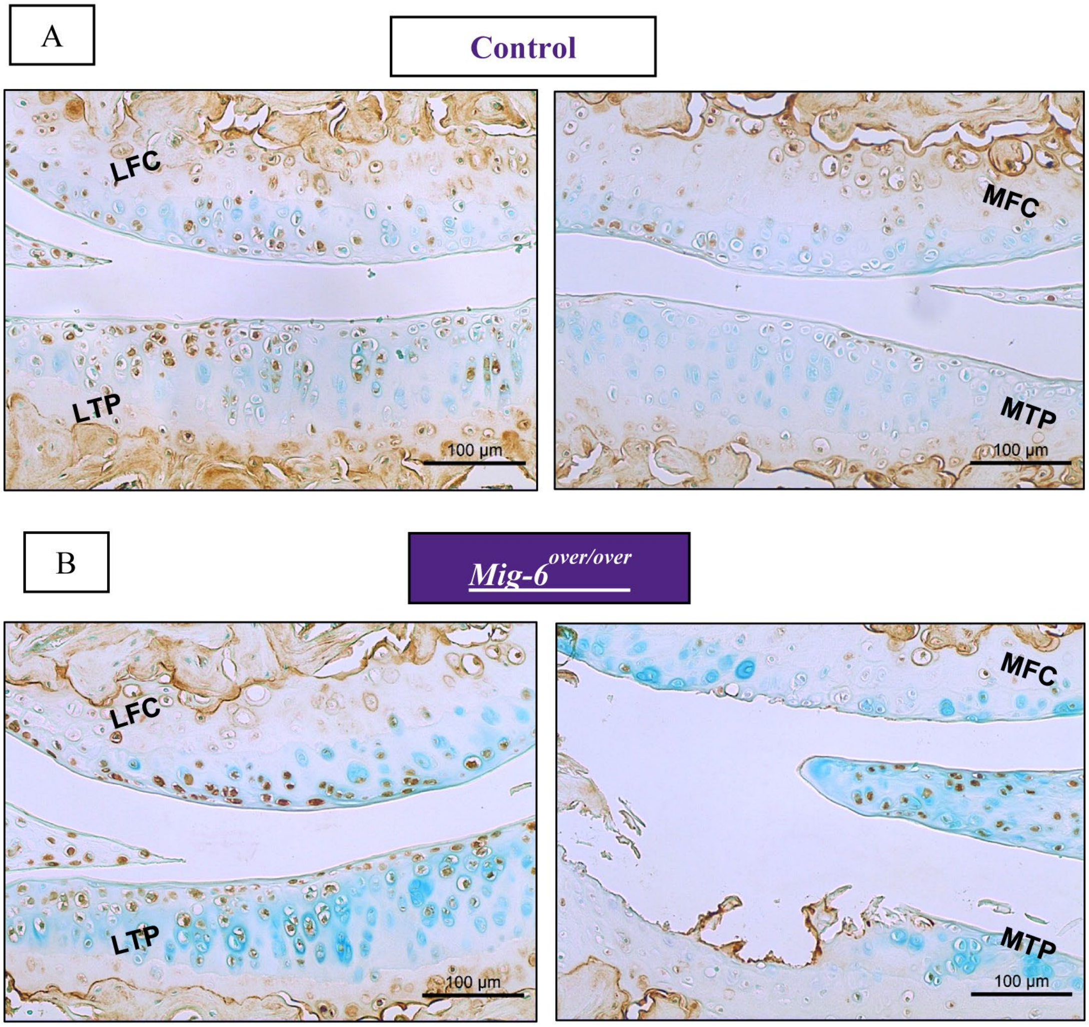

Supplementary Figure 13) 12 month-old cartilage specific Mig-6 overexpressing mice show similar pattern of MMP13 as the control mice. Representative immunohistochemistry of matrix metalloproteinase 13 (MMP13) for Mig-6 overexpression mice at 12 months control **(A)** and cartilage specific Mig-6 overexpression **(B).** N=5 mice/genotyping. LFC = lateral femoral condyle, LTP = lateral tibial plateau, MFC = medial femoral condyle and MTP = medial tibial plateau. Scale bar = 100µm.
